# A real-world study of adverse event profiles associated with the four key components of antibody–drug conjugates based on the FAERS database

**DOI:** 10.3389/fphar.2026.1702195

**Published:** 2026-02-13

**Authors:** Shuning Zhang, Chun Ye, Jiaxuan Zhu, Miao Wang, Yanyan Xu

**Affiliations:** 1 Department of Pharmacy, the Fifth Affiliated Hospital, Wenzhou Medical University, Lishui, Zhejiang, China; 2 School of Pharmacy, Hangzhou Medical College, Hangzhou, Zhejiang, China

**Keywords:** antibody–drug conjugates, component type-specific toxicity, drug-to-antibody ratio, food and drug administration adverse event reporting system, hepatobiliary toxicity, ocular toxicity, pharmacovigilance

## Abstract

**Background:**

Antibody–drug conjugates (ADCs) exhibit structurally driven and highly heterogeneous toxicities, yet most pharmacovigilance studies remain drug-centered and lack systematic component-level evaluation. This study provides a comprehensive real-world safety assessment of FDA-approved ADCs using a component-based analytical framework.

**Methods:**

All adverse event (AE) reports for 14 FDA-approved ADCs (2004–2024) were extracted from the Food and Drug Administration (FDA) Adverse Event Reporting System (FAERS). ADCs and AEs were included when positive signals were simultaneously detected by four disproportionality methods (reporting odds ratio (ROR), proportional reporting ratio (PRR), information component (IC), and empirical Bayes geometric mean (EBGM)) under a multi-method consensus criterion (MCC). AEs were classified using MedDRA at the preferred term (PT) and system organ class (SOC) levels. Signal prioritization was performed by ranking ROR values within each key component type—antibody subtype, linker type, payload category, and drug-to-antibody ratio (DAR) value class, with the top 20 signals designated as “strong signals.” False discovery rate (FDR) correction was applied for sensitivity analysis.

**Results:**

A total of 52,699 positive PT-level AE signals were included across 13 ADCs. Distinct toxicity signatures were observed across component types: IgG1-based ADCs demonstrated prominent ocular and corneal toxicity and treatment-related secondary lymphoma, whereas IgG4-based ADCs were dominated by hepatobiliary injury, vascular toxicity, bone marrow suppression, and severe infections. ADCs with cleavable linkers showed toxicity patterns enriched for treatment-related secondary lymphoma, pulmonary toxicity, and infections, while non-cleavable linker ADCs showed a highly distinctive pattern dominated by ocular toxicity, particularly corneal epithelial and stromal injury. DNA-damaging payload ADCs exhibited severe systemic toxicities, including hepatobiliary toxicity, vascular injury, interstitial lung disease, serious infections, and bone marrow suppression. In contrast, microtubule inhibitor payloads mainly produced ocular and corneal toxicity, treatment-related secondary lymphoma, and local hemorrhagic events. DAR 3–5 ADCs were highly enriched for ocular toxicity and treatment-related secondary lymphoma; DAR < 3 ADCs were characterized by hepatobiliary injury and infection-dominant patterns, and DAR > 5 ADCs demonstrated a heterogeneous multi-organ toxicity profile spanning metabolic, hepatic, pulmonary, infectious, neurological, vascular, and ocular domains. All MCC-identified PT-level AE signals remained significant after FDR correction.

**Conclusion:**

This large-scale real-world evaluation reveals clear component type-specific toxicity signatures among ADCs, supporting more targeted safety monitoring and informing rational ADC design.

## 1 Introduction

Malignant tumors remain the second leading cause of death worldwide, accounting for nearly 10 million deaths each year (Bray et al., 2024). Antibody-drug conjugates (ADCs) are a class of targeted anticancer therapeutics composed of a monoclonal antibody, a linker, and a cytotoxic payload. With the rapid advancement of precision oncology, ADCs have emerged as the main therapeutic drugs. By linking a tumor-targeting monoclonal antibody to a highly potent cytotoxic payload through a specialized linker, ADCs enable targeted delivery of cytotoxic agents to tumor sites, thereby significantly improving the therapeutic index compared with conventional chemotherapy (Fu et al., 2022). Since the approval of gemtuzumab ozogamicin in 2000, more than a dozen ADCs have entered clinical practice for hematologic malignancies and multiple solid tumors, including breast, lung, cervical, gastric, and prostate cancers (Gogia et al., 2023). Despite their therapeutic advantages, ADCs are associated with complex and heterogeneous adverse event (AE) profiles, including hematologic, pulmonary, hepatic, cardiac, and ocular toxicities (Woodford et al., 2025; Koganemaru and Shitara, 2021; Tarantino et al., 2021; Soares et al., 2023; Reike et al., 2024). These toxicities are strongly influenced by the four key components of ADCs—antibody, linker, payload, and drug-to-antibody ratio (DAR)—each of which can affect pharmacokinetics, payload release, and organ-specific toxicity. As the clinical indications and patient exposure to ADCs continue to expand, understanding their real-world safety profiles has become an urgent priority for clinicians and regulatory authorities.

Most of the available safety data on ADCs originate from pivotal clinical trials or post-marketing studies. However, these investigations typically focus on the toxicity of a single ADC or a component. For example, peripheral neuropathy is well recognized in monomethyl auristatin E (MMAE)-containing ADCs (Best et al., 2021), while trastuzumab deruxtecan is associated with interstitial lung disease (ILD) or pneumonitis (Saura et al., 2024). Such drug-level safety assessments limit the ability to determine how specific components contribute to characteristic toxicity patterns across different ADCs. Moreover, emerging real-world evidence has revealed discrepancies between clinical trial findings and post-marketing surveillance. In the DESTINY-Breast01 trial, the incidence of trastuzumab deruxtecan-related ILD was 15.8%, whereas post-marketing reports from Japan documented a higher rate of 27.1% (Cortés et al., 2022). Similar inconsistencies in hematologic toxicity have been observed in observational studies involving polatuzumab vedotin-based treatment (Sehn et al., 2020; Argnani et al., 2022). These findings highlight the complexity of ADC safety in routine clinical practice and underscore the need to investigate ADC-related AEs in larger and more heterogeneous populations. In addition, current evidence often provides only a fragmented view of ADC safety, and the Food and Drug Administration (FDA) Adverse Event Reporting System (FAERS) lacks a systematic “component-specific toxicity” mapping framework. Comprehensive evaluations integrating all four key components of ADCs to explain component-specific toxicity differences remain scarce. Clinicians urgently require component-level real-world evidence to better understand the safety profiles of ADCs and guide proactive risk management before treatment initiation.

To address these limitations, the present study aims to perform a comprehensive, component-oriented safety evaluation of all marketed ADCs using real-world evidence. Specifically, we assess how the four key components—antibody subtype, linker cleavability type, payload category, and DAR value class—shape AE profiles in clinical practice. By moving beyond single-drug analyses, this framework seeks to generate mechanistic insights that directly inform clinical decision-making and the development of future ADCs.

**FIGURE 1:**
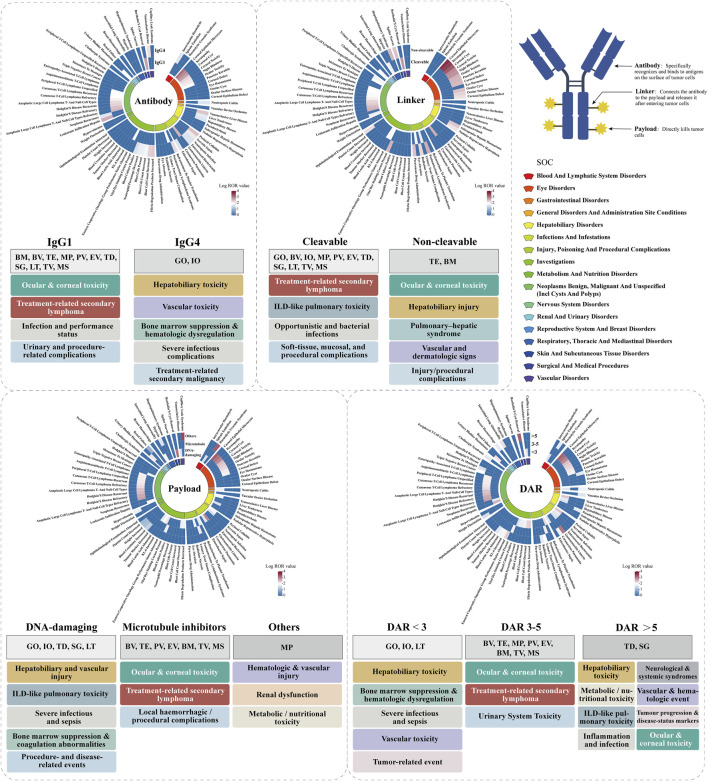
Component-specific safety landscape of FDA-approved ADCs identified from FAERS (2004-2024).

In this study, we utilized the FAERS to conduct a large-scale pharmacovigilance analysis of 13 marketed ADCs from 2004 to 2024. Stratified disproportionality analyses were performed to identify component-specific AE signals across organ systems, with particular focus on hematologic, pulmonary (including ILD), hepatic, neurologic, and ocular toxicities—key safety concerns highlighted in previous ADC research (Woodford et al., 2025). This study provides (i) a systematic real-world evaluation linking the key component features of ADCs to their toxicity patterns; (ii) identification of component-driven safety signals, including ocular toxicity, ILD, hepatotoxicity, and neurotoxicity; and (iii) clinically relevant evidence to support personalized ADC selection and post-marketing safety monitoring (Figure 1). Overall, our findings provide mechanistic and clinically meaningful insights into how ADC components influence their real-world safety profiles.

## 2 Materials and methods

### 2.1 Selection of ADCs and data sources

By reviewing FDA drug approval announcements and product labeling information, we compiled a list of the 14 ADCs approved by the FDA as of 31 December 2024 (Supplementary Table S1): gemtuzumab ozogamicin (GO), brentuximab vedotin (BV), ado-trastuzumab emtansine (TE), inotuzumab ozogamicin (IO), moxetumomab pasudotox-tdfk (MP), polatuzumab vedotin (PV), enfortumab vedotin (EV), fam-trastuzumab deruxtecan-nxki (TD), sacituzumab govitecan (SG), belantamab mafodotin (BM), loncastuximab tesirine (LT), tisotumab vedotin (TV), mirvetuximab soravtansine (MS), and datopotamab deruxtecan (DD).

For the present analysis, we included ADCs that met the following criteria (Bray et al., 2024): FDA approved for marketing during the study period (2004–2024); and (Fu et al., 2022) the ADC must have had at least one adverse event reported in FAERS between 2004 and 2024 that generated a positive signal across all four disproportionality methods: reporting odds ratio (ROR), proportional reporting ratio (PRR), information component (IC), and empirical Bayes geometric mean (EBGM). ADCs that were excluded from our analysis, along with the reasons for exclusion, are listed in Supplementary Table S1. For each included ADC, we extracted key drug characteristics from FDA prescribing information and approval documents, including generic name, brand name, approval date, approved indications, antibody subtype (IgG1 vs. IgG4), linker type (cleavable vs. non-cleavable), payload category (DNA-damaging agent, microtubule inhibitor, and other), and drug-to-antibody ratio (DAR < 3, 3–5, >5).

**FIGURE 2:**
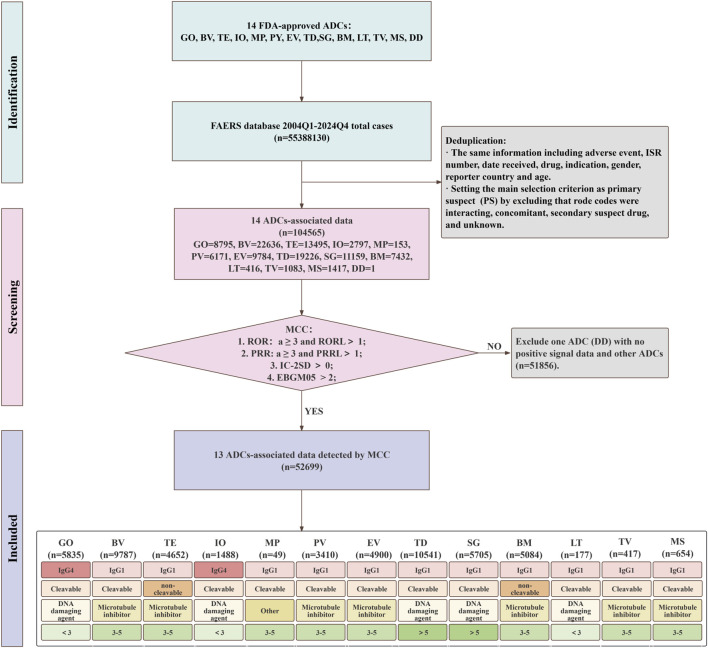
The process of selecting ADC-related AEs from FAERS database and stratified analysis workflow.

We retrieved all AE reports for the 14 ADCs from the FAERS database for the period 2004Q1 to 2024Q4 using the OpenVigil 2.1 interface. Data cleaning and deduplication procedures were performed in accordance with the FDA’s official guidance (Yang et al., 2024). Adverse events were categorized using the Medical Dictionary for Regulatory Activities 27.0 at both the preferred term (PT) and system organ class (SOC) levels. Because the data were publicly available, no ethics approval was required. The complete screening workflow is illustrated in Figure 2.

### 2.2 Signal detection and disproportionality analysis

Disproportionality analyses were performed to evaluate the association between each ADC key component type and specific AEs. For every component type–AE pair, a 2 × 2 contingency table was constructed (Supplementary Table S2), and disproportionality was quantified to identify potential pharmacovigilance signals. We applied a multi-method consensus criteria (MCC) approach for signal detection to ensure robustness. An AE was considered a positive signal only if it simultaneously met the predefined thresholds for all four algorithms (ROR, PRR, IC, and EBGM). The computational formulas and threshold criteria for each method are summarized in Supplementary Table S3.

### 2.3 Signal prioritization

To further prioritize signals with potential major clinical implications, we incorporated the magnitude of statistical association as a key criterion, in accordance with methodological guidance on signal prioritization in pharmacovigilance research (Public Policy Committee and International Society of Pharmacoepidemiology, 2016; Heads of Medicines Agencies and European Medicines Agency, 2017). After applying the MCC to identify statistically significant signals, we ranked all positive signals within each ADC key component type by their ROR. Signals with ROR values within the top 20 for that component type were classified as “strong signals” and prioritized for further evaluation and discussion. This approach aligns with regulatory recommendations that emphasize both statistical robustness and the potential clinical impact of emerging safety signals. For the payload subclass “others,” in which the total number of positive AE signals was small (n = 8), all signals were included in the final analysis to ensure a comprehensive assessment.

### 2.4 Statistical analysis

In the baseline analysis of ADC key components, we used Cramer’s V coefficient and *p*-value to assess intergroup differences in categorical variables. This method is well-suited for large sample sizes. When *p*-value >0.05 and Cramer’s V is near zero, it suggests a weak correlation that may have occurred by chance and is therefore considered unreliable. The Shapiro–Wilk test was used to assess the normality of continuous variables. Normally distributed results were presented as the mean ± SD and compared using the independent t-test, whereas non-normally distributed results are presented as median (Q1, Q3) and evaluated using the Mann–Whitney U test. Categorical data are presented as n (%) and compared using the chi-square or Fisher’s exact test. A two-sided *p* < 0.05 indicated significance. Onset time (OT) was defined as the interval between ADC treatment initiation and the occurrence of an AE. Missing data, erroneous data, and implausible dates (e.g., negative values) were excluded.

To control the risk of false-positive findings arising from multiple comparisons, we performed a false discovery rate (FDR) correction as a sensitivity analysis. For each combination of an ADC key component type and an AE, we calculated the *p*-value for the association using Fisher’s exact test. All *p*-values were then adjusted using the Benjamini–Hochberg procedure, with statistical significance defined as *q* < 0.05. The AE positive signal detection relied on MCC, whereas the FDR-adjusted results were used to evaluate the robustness of these positive signals.

## 3 Results

### 3.1 General characteristics

From FAERS, a total of 55,388,130 AE reports (2004–2024) were initially retrieved. Across the 13 included ADCs, a total of 35,158 AE reports were identified, involving 1,045,654 PT-level AEs. Following the exclusion of 51,857 AE reports that did not comply with the criteria of MCC, 52,699 AE reports were retained for final analysis (Figure 2). As shown in Supplementary Table S4, TD had the maximum number of case reports (n = 7,497) and the second largest total number of AE reports (n = 19,226). MP had the fewest case reports (n = 58) and the lowest number of AE reports (n = 153). This is primarily because MP was withdrawn from the market in August 2023.

**FIGURE 3:**
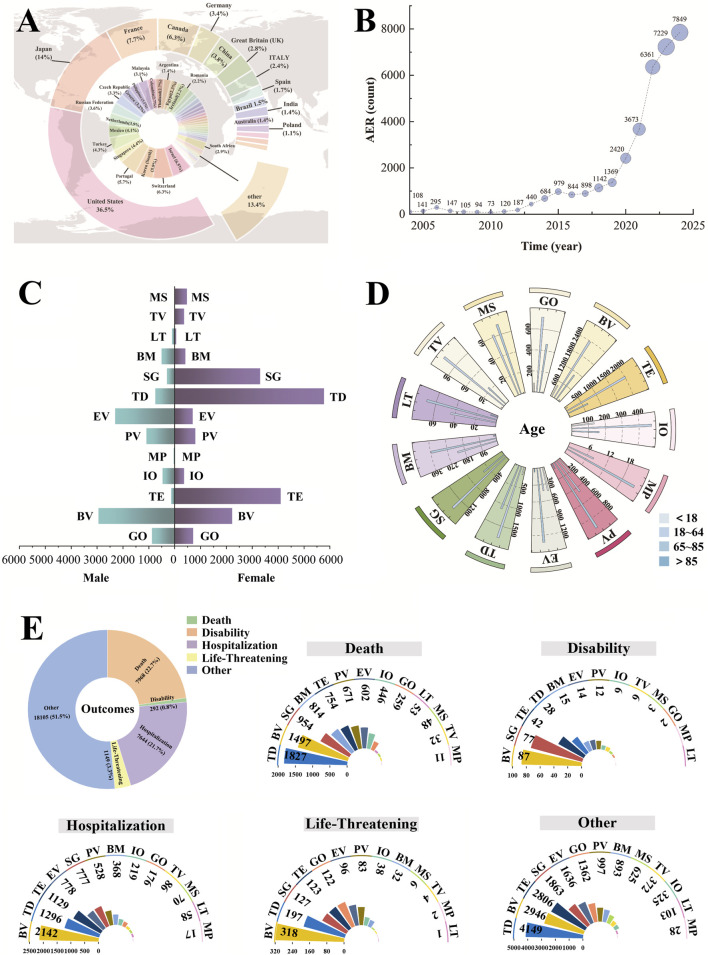
Characteristics of reports associated with ADCs extract from 2004 to Q4 of 2024.

As shown in Figure 3A, the top five countries in terms of the number of global AE reports were as follows: the United States (36.5%), Japan (14%), France (7.7%), Canada (6.3%), and China (3.8%). Figure 3B shows the upward trend in the total number of ADC-related AEs from 2004 to 2024. The number of AEs submitted has grown rapidly since 2020. Figure 3C illustrates sex-specific differences in ADC-related AEs, with female participants accounting for 54.9% (19,306/35,158) and male participants for 27.2% (9,560/35,158). Across TE, TD, SG, TV, and MS, female participants constituted a markedly larger proportion of cases, with 4,102 (84.5%), 5,775 (77.0%), 3,306 (87.0%), 363 (72.6%), and 476 (63.3%) cases, respectively, far outnumbering male participants. Regarding age distribution (Figure 3D), most AE reports were concentrated in the 18–64 and 65–85 age groups. However, there are more reports of IO and EV in the age group under 18, accounting for 13.2% and 13.7%, respectively. Serious adverse outcomes associated with ADC-related AEs included hospitalization, disability, life-threatening conditions, death, and unknown outcomes (Figure 3E). Excluding other outcomes (51.5%), the proportions of patients experiencing hospitalization and death were 21.7% and 22.7%, respectively. Importantly, the majority of AE reports (n = 26,848, 76.4%) were reported by physicians, nurses, and pharmacists, underscoring the reliability and clinical relevance of the data.

### 3.2 Signal of SOC

**FIGURE 4:**
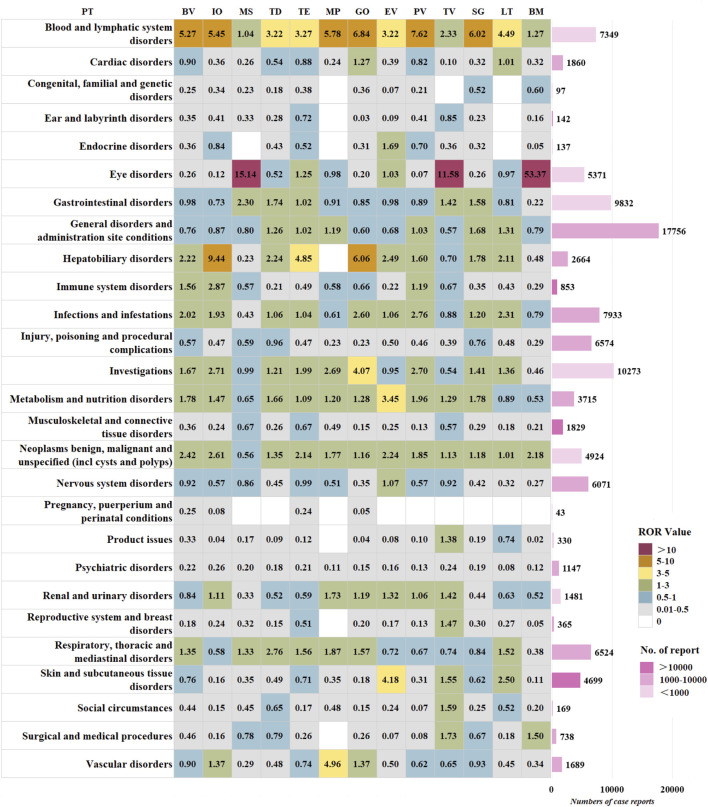
Signal strength of AE reports of ADCs at the SOC level.

Figure 4 displays the distribution of signal strength across different SOC levels for the 13 ADCs. The most frequently reported SOCs were general disorders and administration site conditions, investigations, and gastrointestinal disorders. The top three SOCs by signal strength were eye disorders, blood and lymphatic system disorders, and hepatobiliary disorders. Within the SOC of eye disorders, the strongest signals were observed for BM (ROR = 53.37), MS (ROR = 15.14), and TV (ROR = 11.58). All ADCs were associated with blood and lymphatic system disorders, with the strongest signals observed for PV (ROR = 7.62), GO (ROR = 6.84), and SG (ROR = 6.02), while BM (ROR = 1.27) and MS (ROR = 1.04) showed relatively weaker effects. For hepatobiliary disorders, the stronger signals were detected with IO (ROR = 9.44), GO (ROR = 6.06), and TE (ROR = 4.85).

### 3.3 AE signals at PT level

A total of 52,699 positive PT-level AE signals were detected across 13 ADCs, distributed as follows and as shown in Figure 2: GO (5,835), BV (9,787), TE (4,652), IO (1,488), MP (49), PV (3,410), EV (4,900), TD (10,541), SG (5,705), BM (5,084), LT (177), TV (417), and MS (654). We analyzed all detected signals, with particular focus on the top 20 AEs sorted by frequency and signal strength, as detailed in Supplementary Tables S5 and S6. Several AEs were identified that were not listed in their instructions, such as perineal cellulitis with GO (ROR = 403.34), axonal and demyelinating polyneuropathy with BV (ROR = 142.27), spider nevus with TE (ROR = 446.96), graft-versus-host disease in the liver with IO (ROR = 126.31), increased blood creatinine with MP (ROR = 24.58), lymphoma transformation (ROR = 170.01), cytomegalovirus hepatitis (ROR = 84.18), and heat illness (ROR = 44.74) with PV, KL-6 elevation (ROR = 324.74) and mechanical ileus (ROR = 70.58) with EV, splenic embolism with TD (ROR = 90.30), neutropenic sepsis with SG (ROR = 30.95), corneal vital dye staining with BM (ROR = 1,038.97), intercepted medication error with LT (ROR = 90.47), vaginal hemorrhage with TV (ROR = 15.59), and brain fog with MS (ROR = 16.53).

### 3.4 Stratified analysis of antibody type

The 13 ADCs were categorized into two groups based on their antibody subtype: IgG1 and IgG4. After excluding unspecified cases, the IgG1 group comprised a higher proportion of female participants (56.61%), whereas the IgG4 group included more male participants (45.69%). The highest incidence of AEs was in individuals aged 18–64 years, accounting for 27.61% in the IgG1 group and 40.24% in the IgG4 group. In both groups, most reported adverse events were serious AEs, and detailed distributions are presented in Table 1. After excluding AE reports with missing and abnormal OT data, the median of OT for the IgG1 antibody was 23 days (Reike et al., 2024; Sun et al., 2024), which was much longer than the IgG4 antibody at 9 days (Gogia et al., 2023; Wang et al., 2022). As shown in Supplementary Figure S1, there was a difference in OT between IgG1- and IgG4-based ADCs. The distribution of ADCs across each OT period also differed.

**TABLE 1:** Baseline characteristics of IgG1 and IgG4 antibodies of ADCs.

Characteristic	IgG1 (n = 32,203)	IgG4 (n = 2,955)	Total (n = 35,158)	Statistics
Gender, n (%)				Cramer’s V 0.107, *p* < 0.001
Male	8,210 (25.49)	1,350 (45.69)	9,560 (27.19)	​
Female	18,229 (56.61)	1,077 (36.45)	19,306 (54.91)	​
Unspecified	5,764 (17.90)	528 (17.87)	6,292 (17.90)	​
Age (years), n (%)				Cramer’s V 0.107, *p* < 0.001
<18	1,368 (4.25)	222 (7.51)	1,590 (4.52)	​
18–64	8,891 (27.61)	1,189 (40.24)	10,080 (28.67)	​
65–85	6,153 (19.11)	626 (21.18)	6,779 (19.28)	​
>85	280 (0.87)	12 (0.41)	292 (0.83)	​
Unspecified	15,511 (48.17)	906 (30.66)	16,417 (46.69)	​
Median (Q1, Q3)	59 (44, 70)	55 (34, 68)	59 (43, 70)	​
Min, max	0.25, 110	0.20,93	0.20, 110	​
Severity of report, n (%)				Cramer’s V 0.159, *p* < 0.001
Serious	26,204 (81.37)	1,722 (58.27)	27,926 (79.43)	​
Non-serious	5,999 (18.63)	1,233 (41.73)	7,232 (20.57)	​
Outcomes, n (%)				Cramer’s V 0.072, *p* < 0.001
Death	7,263 (22.55)	705 (23.86)	7,968 (22.66)	​
Disability	284 (0.88)	8 (0.27)	292 (0.83)	​
Hospitalization (initial or prolonged)	7,249 (22.51)	395 (13.37)	7,644 (21.74)	​
Life-threatening	989 (3.07)	160 (5.41)	1,149 (3.27)	​
Other serious report	16,418 (50.98)	1,687 (57.09)	18,105 (51.50)	​
Onset time (days)				​
Median (Q1, Q3)	23 (8, 27)	9 (3, 25)	26 (9, 85)	​

The onset time recorded in the database refers to the date when a patient first experiences any AE, rather than a particular AE. When calculating onset time, values less than 0 days were retained after imputing missing data.

Abbreviations: SD, standard deviation; Q1, lower quartile; Q3, upper quartile.

**FIGURE 5:**
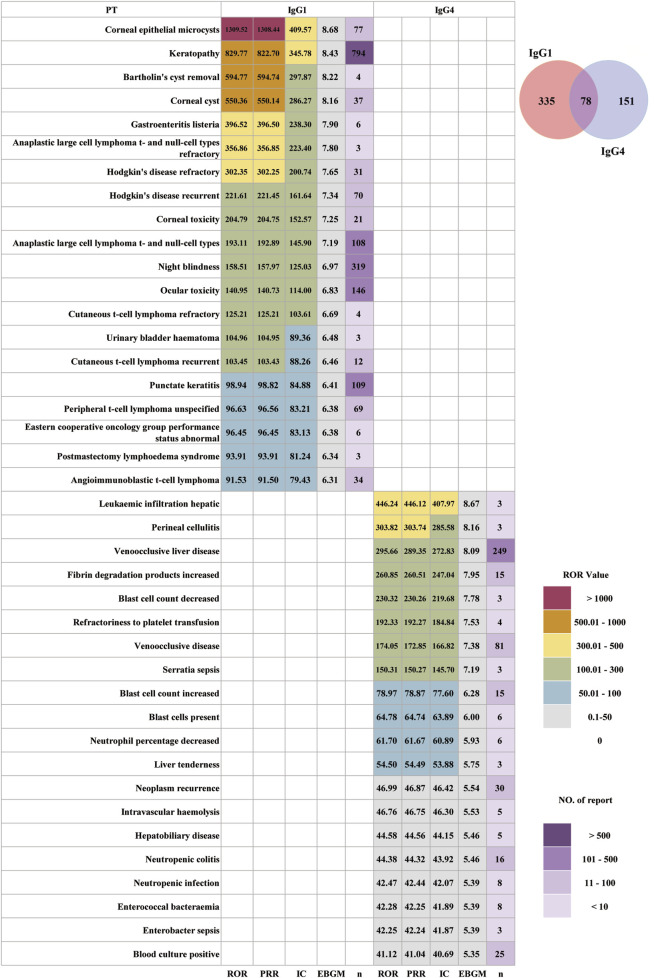
Distribution of AE signal intensity for ADCs with IgG1 and IgG4 antibodies.

Figure 5 shows the PT-level AE signals between the IgG1 and IgG4 groups, revealing clear differences in both the distribution of AEs and signal strength. ADCs with IgG1 antibody were associated with 335 unique AEs, whereas IgG4-based ADCs had 151 unique AEs. The two groups shared 78 AEs. In the IgG1 group, the top 20 strongest signals were predominantly concentrated in ocular and corneal toxicity and treatment-related secondary lymphoma, accompanied by infections, performance status impairment, and urinary- and procedure-related complications. The strongest AE signals included corneal epithelial microcysts (ROR = 1,309.52), keratopathy (ROR = 829.77), Bartholin’s cyst removal (ROR = 594.77), corneal cyst (ROR = 550.36), punctate keratitis (ROR = 98.94), refractory anaplastic large-cell lymphoma (ROR = 356.86), recurrent Hodgkin’s disease (ROR = 221.61), and recurrent cutaneous T-cell lymphoma (ROR = 103.45). For the IgG4 group, the strongest AE signals differed in profile. The strongest signals included leukemic hepatic infiltration (ROR = 446.24), veno-occlusive liver disease (ROR = 295.66), hepatobiliary disease (ROR = 44.58), increased fibrin degradation products (ROR = 260.85), decreased blast cell count (ROR = 230.32), refractoriness to platelet transfusion (ROR = 192.33), *serratia* sepsis (ROR = 150.31), enterococcal bacteremia (ROR = 42.28), and blood culture positivity (ROR = 41.12). In contrast to IgG1-based ADCs, IgG4-based ADCs are characterized primarily by hepatobiliary toxicity, vascular toxicity, bone marrow suppression, hematologic dysregulation, severe infections, and treatment-related secondary malignancies, with minimal occurrence of the highly enriched ocular toxicity observed in IgG1 constructs.

### 3.5 Stratified analysis of linker type

Based on linker cleavage mechanisms, the analysis was stratified into two groups: cleavable and non-cleavable. The proportion of female participants was 54.91%, with 52.48% in the cleavable group and 64.75% in the non-cleavable group, both higher than those of male participants (Table 2). The highest incidence of AEs was observed in individuals aged 18–64 years, accounting for 27.85% of the cleavable group and 31.98% of the non-cleavable group. In both groups, most reported AEs were classified as serious reports, and there were no significant differences in outcomes. After excluding AE reports with missing or abnormal OT data, the median of OT for cleavable linkers was 18 days (7, 57), which was much shorter than that of non-cleavable linkers at 40 days (15, 105). OT between the cleavable and non-cleavable linkers differed. The distribution of 13 ADCs across each OT period also differed, as shown in Supplementary Figure S2.

**TABLE 2:** Baseline characteristics of cleavable and non-cleavable linkers of ADCs.

Characteristic	Cleavable (n = 28,182)	Non-cleavable (n = 6,976)	Total (n = 35,158)	Statistics
Gender, n (%)				Cramer’s V 0.206, *p* < 0.001
Male	8,910 (31.62)	650 (9.32)	9,560 (27.19)	​
Female	14,789 (52.48)	4,517 (64.75)	19,306 (54.91)	​
Unspecified	4,483 (15.91)	1,809 (25.93)	6,292 (17.90)	​
Age (years), n (%)				Cramer’s V 0.110, *p* < 0.001
<18	1,515 (5.38)	75 (1.08)	1,590 (4.52)	​
18–64	7,849 (27.85)	2,231 (31.98)	10,080 (28.67)	​
65–85	5,767 (20.46)	1,012 (14.51)	6,779 (19.28)	​
>85	253 (0.90)	39 (0.56)	292 (0.83)	​
Unspecified	12,798 (45.41)	3,619 (51.88)	16,417 (46.69)	​
Median (Q1, Q3)	59 (41, 71)	58 (49, 67)	59 (43, 70)	​
Min, Max	0.20, 110	0.25, 94	0.20, 110	​
Severity of report, n (%)				Cramer’s V 0.022, *p* < 0.001
Serious	22,260 (78.99)	5,666 (81.22)	27,926 (79.43)	​
Non-serious	5,922 (21.01)	1,310 (18.78)	7,232 (20.57)	​
Outcomes, n (%)				Cramer’s V 0.031, *p* < 0.001
Death	6,400 (22.71)	1,568 (22.48)	7,968 (22.66)	​
Disability	235 (0.83)	57 (0.82)	292 (0.83)	​
Hospitalization (initial or prolonged)	6,147 (21.81)	1,497 (21.46)	7,644 (21.74)	​
Life-threatening	994 (3.53)	155 (2.22)	1,149 (3.27)	​
Other serious report	14,406 (51.12)	3,699 (53.02)	18,105 (51.50)	​
Onset time (days)				​
Median (Q1, Q3)	18 (7, 57)	40 (15, 105)	26 (9, 85)	​

**FIGURE 6:**
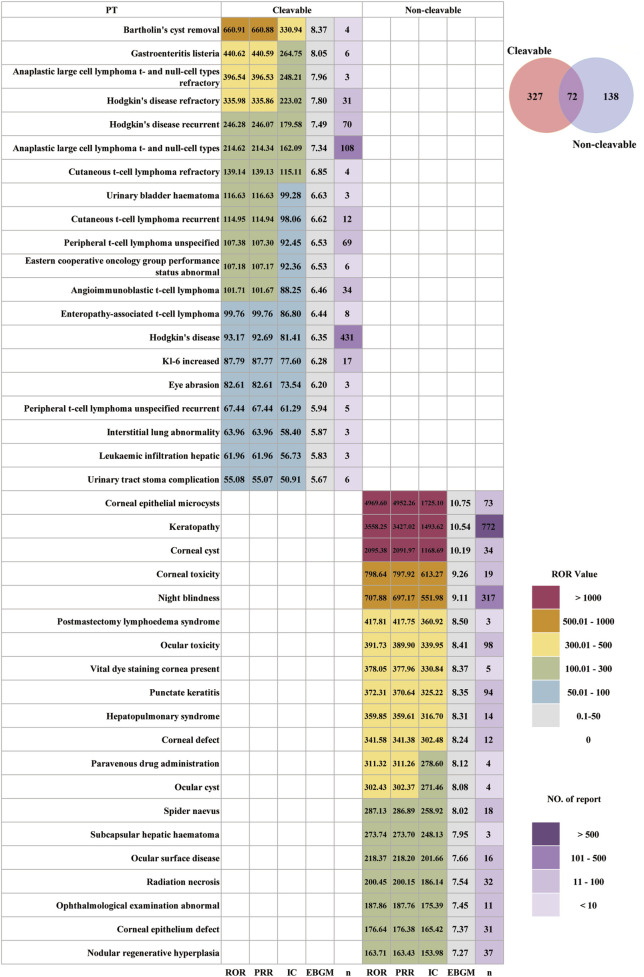
Distribution of AE signal strength in cleavable and non-cleavable linker ADCs.

Figure 6 shows the results of AE signals at PT-level between the two linker-type groups detected by MCC. ADCs with non-cleavable linkers were associated with 138 unique AEs, whereas cleavable-linker ADCs had 327 unique AEs, with 72 AEs shared between the two groups. Cleavable linker ADCs exhibit a toxicity profile characterized by treatment-related secondary lymphoma, ILD-like pulmonary toxicity, opportunistic and bacterial infections, and soft-tissue, mucosal, and procedural complications. The top 20 PT-level positive signals were mainly focused on (Bray et al., 2024) Bartholin’s cyst removal (ROR = 660.91) and gastroenteritis *listeria* (ROR = 440.62), which showed the highest ROR values (Fu et al., 2022). Multiple T-cell lymphoma-related PTs included anaplastic large-cell lymphoma (ROR = 396.54), cutaneous T-cell lymphoma (ROR = 139.14), and angioimmunoblastic T-cell lymphoma (ROR = 101.71). Among these signals, Hodgkin’s disease has the highest number of reports (n = 431). In contrast, non-cleavable linker ADCs demonstrate a distinctly ocular toxicity-dominated profile. The ocular structures, such as corneal epithelium, stromal layers, and visual function, are the most prominently affected targets. These ocular effects represent the most clinically consequential toxicities. Hepatobiliary injury and pulmonary-hepatic syndrome occur as secondary but relevant toxicities. The strong signals included corneal epithelial microcysts (ROR = 4,969.60), keratopathy (ROR = 3,558.25), corneal cyst (ROR = 2095.38), corneal toxicity (ROR = 798.64), night blindness (ROR = 707.88), punctate keratitis (ROR = 372.31), hepatopulmonary syndrome (ROR = 359.85), subcapsular hepatic hematoma (ROR = 273.74), and nodular regenerative hyperplasia (ROR = 163.71). Among these signals, the incidence rates of keratopathy and corneal keratopathy were very high, with 772 and 317 reported cases, respectively. This pattern demonstrates a distinct divergence in AE distribution and signal strength profiles between the two linker types.

### 3.6 Stratified analysis of payload type

Based on the payload type, ADCs were categorized into three groups: DNA-damaging agents, microtubule inhibitors, and others. Table 3 showed that in the DNA-damaging agent and microtubule inhibitor groups, the proportion of female patients exceeded that of male patients, at 70.65% and 43.99%, respectively. After excluding unspecified cases, the highest incidence of AEs was observed in individuals aged 18–64 years, accounting for 30.58% in the DNA-damaging agent group and 27.38% in the microtubule inhibitor group. In contrast, for ADCs with other payloads, the highest incidence occurred in the 65–85 age group (37.93%). Across all three categories, most reported AEs were classified as serious. The most common outcomes were death and hospitalization, which included initial or prolonged hospitalization. After excluding AE reports with missing or abnormal OT data, the median of OT in the group of DNA-damaging agents was 57 days (45, 68), much longer than that of the other two groups. The median OT for the microtubule inhibitor group and the other group was 24 days (8, 72) and 4 days (Gogia et al., 2023; Kloehn et al., 2022), respectively. As shown in Supplementary Figure S3, the OT was different among the payload type groups. The distribution of ADCs across each OT period also differed.

**TABLE 3:** Baseline characteristics of different payloads of ADCs.

Characteristic	DNA-damaging agents (n = 14,465)	Microtubule inhibitors (n = 20,635)	Others (n = 58)	Total (n = 35,158)	Statistics
Gender, n (%)					Cramer’s V 0.188, *p* < 0.001
Male	2,506 (17.32)	7,014 (33.99)	40 (68.97)	9,560 (27.19)	​
Female	10,219 (70.65)	9,077 (43.99)	10 (17.24)	19,306 (54.91)	​
Unspecified	1,740 (12.03)	4,544 (22.02)	8 (13.79)	6,292 (17.90)	​
Age (years), median (Q1, Q3)					Cramer’s V 0.081, *p* < 0.001
<18	418 (2.89)	1,172 (5.68)	0	1,590 (4.52)	​
18–64	4,423 (30.58)	5,649 (27.38)	8 (13.79)	10,080 (28.67)	​
65–85	2,315 (16.00)	4,442 (21.53)	22 (37.93)	6,779 (19.28)	​
>85	53 (0.37)	239 (1.16)	0	292 (0.83)	​
Unspecified	7,256 (50.16)	9,133 (44.26)	28 (48.28)	16,417 (46.69)	​
Median (Q1, Q3)	57 (45, 68)	60 (41, 71)	72 (65, 78)	57 (43, 70)	​
Min, max	0.20, 95	0.25, 110	44, 80	0.20, 110	​
Severity of report, n (%)					Cramer’s V 0.106, *p* < 0.001
Serious	10,749 (74.31)	17,135 (83.04)	42 (72.41)	27,926 (79.43)	​
Non-serious	3,716 (25.69)	3,500 (16.96)	16 (27.59)	7,232 (20.57)	​
Outcomes, n (%)					Cramer’s V 0.062, *p* < 0.001
Death	3,539 (24.47)	4,418 (21.41)	11 (18.97)	7,968 (22.66)	​
Disability	113 (0.78)	179 (0.87)	0	292 (0.83)	​
Hospitalization (initial or prolonged)	2,526 (17.46)	5,101 (24.72)	17 (29.31)	7,644 (21.74)	​
Life-threatening	485 (3.35)	662 (3.21)	2 (3.45)	1,149 (3.27)	​
Other serious report	7,802 (53.94)	10,275 (49.79)	28 (48.28)	18,105 (51.50)	​
Onset time (days)	​				​
Median (Q1, Q3)	57 (45, 68)	24 (8, 72)	4 (3, 28)	26 (9, 85)	​

**FIGURE 7:**
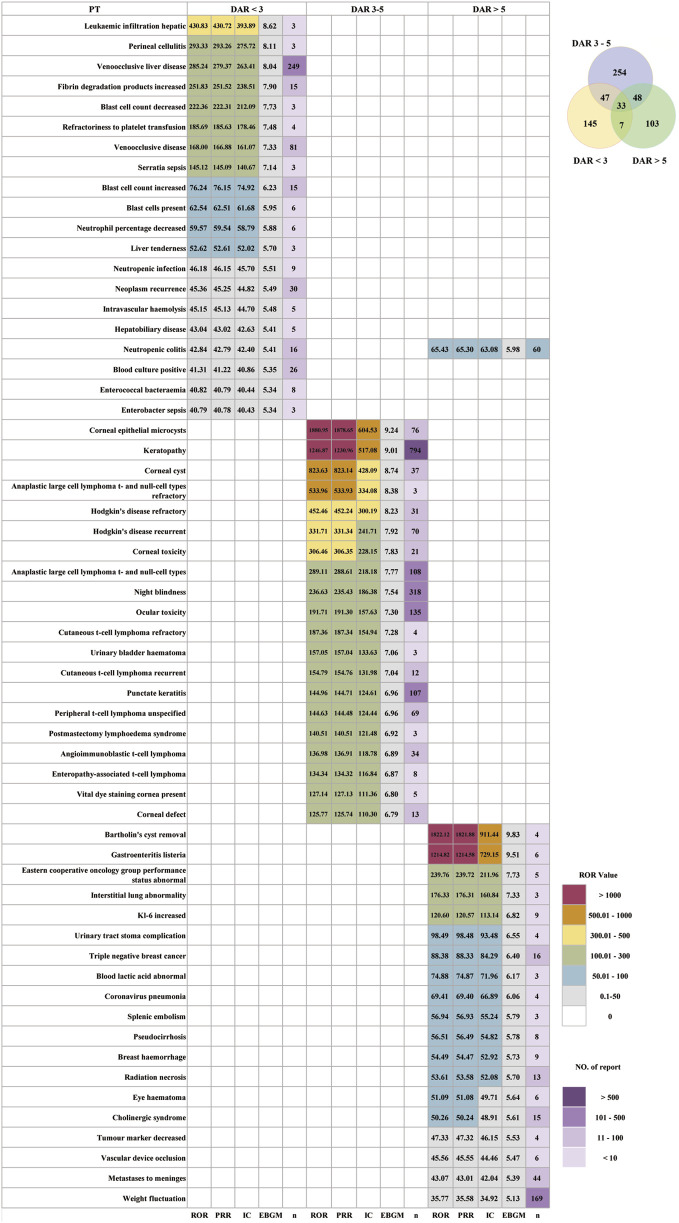
Distribution of AE signal strength for different payloads of ADCs.

Figure 7 illustrated clear differences in the distribution and strength of PT-level AE signals across the three payload types. The DNA-damaging agent group showed 154 unique AEs, the microtubule inhibitor group had 270, and the “others” payload group had 5. DNA-damaging payload ADCs show a highly systemic and severe toxicity profile. Their dominant features include hepatobiliary and vascular injury, ILD-like pulmonary toxicity, severe infections and sepsis, bone marrow suppression and coagulation abnormalities, and procedure- and disease-related events. Strong signals included Bartholin’s cyst removal (ROR = 1,304.28), gastroenteritis *listeria* (ROR = 869.56), interstitial lung abnormality (ROR = 126.22), leukemic infiltration hepatic (ROR = 122.27), KL-6 increased (ROR = 86.32), neutropenic colitis (ROR = 59.89), refractoriness to platelet transfusion (ROR = 52.70), and veno-occlusive disease (ROR = 48.07). Microtubule inhibitor payload ADCs exhibit a toxicity profile characterized by highly enriched ocular and corneal toxicity, treatment-related secondary lymphoma, and local hemorrhagic and procedural complications. The ROR values of AE signals related to ocular and corneal toxicity are as follows: corneal epithelial microcysts (ROR = 1885.63), keratopathy (ROR = 1,250.01), corneal cyst (ROR = 825.68), corneal toxicity (ROR = 307.22), night blindness (ROR = 237.22), ocular toxicity (ROR = 192.19), and corneal defect (ROR = 126.08). The “others” payload type showed fewer signals but demonstrated a highly systemic toxicity pattern centered on endothelial injury as the core pathological feature. The top three strong signals were capillary leak syndrome (ROR = 2,955.17), hemolytic uremic syndrome (ROR = 2,108.33), and hyperkalemia (ROR = 61.03).

### 3.7 Stratified analysis of DAR value

Based on the DAR values, ADCs were categorized into three groups: DAR < 3, DAR 3-5, and DAR > 5. As shown in Table 4, after excluding unspecified cases, the DAR < 3 group exhibited a higher ratio of male patients (45.90%), whereas the groups of DAR 3-5 and DAR > 5 showed a predominance of female patients, accounting for 43.91% and 80.40%, respectively. The highest incidence of AEs was consistently 18–64 years across all three DAR categories, with proportions of 38.90%, 27.34%, and 28.24%, respectively. Most of the AE reports in all groups were serious, with a higher incidence of outcomes categorized as death. After excluding AE reports with missing or abnormal OT data, the median in the DAR < 3 group was 10 days (Woodford et al., 2025; Kantarjian et al., 2017), much shorter than that of the other two groups. The median OT for the DAR 3–5 and DAR > 5 groups was 23 days (8, 72) and 21 days (8, 79), respectively. The OT differed among groups with different DAR values. As shown in Supplementary Figure S4, the distribution of ADCs across each OT period also differed.

**TABLE 4:** Baseline characteristics of different DAR values of ADCs.

Characteristic	DAR < 3 (n = 3,170)	DAR 3–5 (n = 20,693)	DAR > 5 (n = 11,295)	Total (n = 35,158)	Statistics
Gender, n (%)		​			Cramer’s V 0.257, *p* < 0.001
Male	1,455 (45.90)	7,054 (34.09)	1,051 (9.31)	9,560 (27.19)	​
Female	1,138 (35.90)	9,087 (43.91)	9,081 (80.40)	19,306 (54.91)	​
Unspecified	577 (18.20)	4,552 (22.00)	1,163 (10.30)	6,292 (17.90)	​
Age (years), median (Q1, Q3)		​			Cramer’s V 0.123, *p* < 0.001
<18	222 (7.00)	1,172 (5.66)	196 (1.74)	1,590 (4.52)	​
18–64	1,233 (38.90)	5,657 (27.34)	3,190 (28.24)	10,080 (28.67)	​
65–85	695 (21.92)	4,464 (21.57)	1,620 (14.34)	6,779 (19.28)	​
>85	19 (0.60)	239 (1.15)	34 (0.30)	292 (0.83)	​
Unspecified	1,001 (31.58)	9,161 (44.27)	6,255 (55.38)	16,417 (46.69)	​
Median (Q1, Q3)	55 (35, 68)	60 (41, 71)	58 (48, 68)	59 (43, 70)	​
Min, max	0.20, 93	0.25, 110	0.30, 95	0.20, 110	​
Severity of report, n (%)		​			Cramer’s V 0.168, *p* < 0.001
Serious	1,865 (58.83)	17,177 (83.01)	8,884 (78.65)	27,926 (79.43)	​
Non-serious	1,305 (41.17)	3,516 (16.99)	2,411 (21.35)	7,232 (20.57)	​
Outcomes, n (%)					Cramer’s V 0.070, *p* < 0.001
Death	758 (23.91)	4,429 (21.40)	2,781 (24.62)	7,968 (22.66)	​
Disability	8 (0.25)	179 (0.87)	105 (0.93)	292 (0.83)	​
Hospitalization (initial or prolonged)	453 (14.29)	5,118 (24.73)	2,073 (18.35)	7,644 (21.74)	​
Life-threatening	161 (5.08)	664 (3.21)	324 (2.87)	1,149 (3.27)	​
Other serious report	1,790 (56.47)	10,303 (49.79)	6,012 (53.23)	18,105 (51.50)	​
Onset time (days)		​			​
Median (Q1, Q3)	10 (4, 26)	23 (8, 72)	21 (8, 79)	26 (9, 85)	​

**FIGURE 8:**
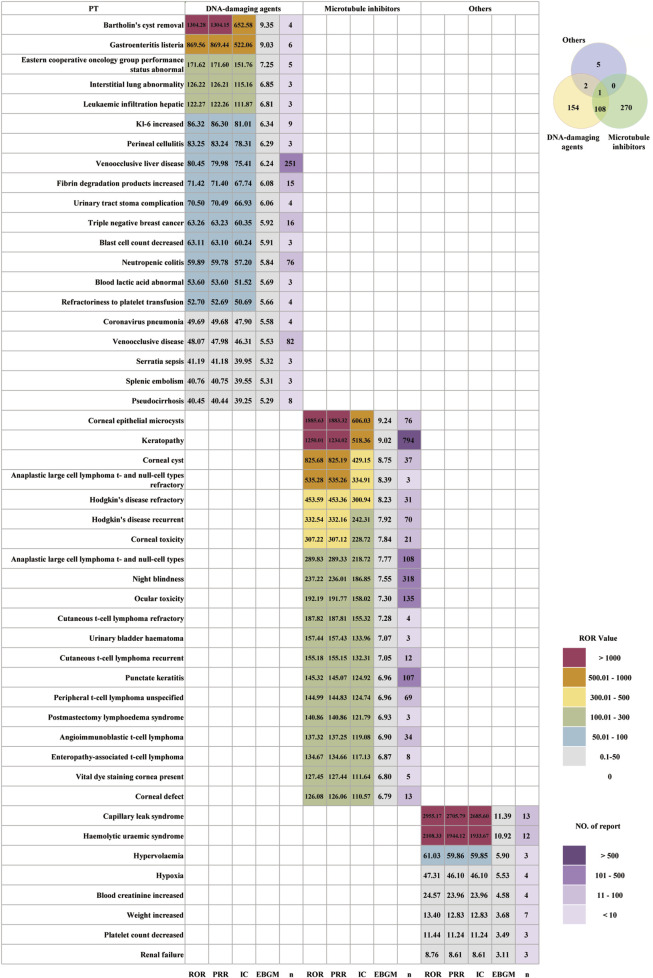
Distribution of AE signal intensity among different drug antibody ratios (DAR) in ADCs.

In Figure 8, the distribution and strength of PT-level AE signals varied significantly across different DAR value groups. ADCs with DAR < 3 had 145 unique AEs, those with DAR 3–5 had 254, and those with DAR > 5 had 103, with 33 AEs shared among all three groups. DAR < 3 ADCs exhibit a toxicity profile dominated by hepatobiliary toxicity, bone marrow suppression, hematologic dysregulation, severe infections and sepsis, vascular toxicity, and tumor-related event patterns. Strong signal PTs included leukemic infiltration of the liver (ROR = 430.83), perineal cellulitis (ROR = 293.33), veno-occlusive liver disease (ROR = 285.24), increased fibrin degradation products (ROR = 251.83), and decreased blast cell count (ROR = 222.36). DAR 3–5 ADCs show toxicity that is highly concentrated in ocular and corneal toxicity, treatment-related secondary lymphoma, and urinary system toxicity. The top-ranked PTs included corneal epithelial microcysts (ROR = 1880.95), keratopathy (ROR = 1,246.87), corneal cysts (ROR = 823.63), refractory anaplastic large-cell lymphoma t- and null-cell types (ROR = 533.96), and refractory Hodgkin’s disease (ROR = 452.46). DAR > 5 ADCs exhibited a highly heterogeneous, multi-organ toxicity pattern involving hepatobiliary toxicity, metabolic/nutritional toxicity, ILD-like pulmonary toxicity, inflammation and infection, neurological and systemic syndromes, vascular and hematologic events, tumor progression and disease-status markers, and ocular toxicity. The highest-ranked PTs included Bartholin’s cyst removal (ROR = 1822.12), gastroenteritis *listeria* (ROR = 1,214.82), Eastern Cooperative Oncology Group performance status abnormal (ROR = 239.76), interstitial lung abnormality (ROR = 176.33), increased KL-6 (ROR = 120.60), urinary tract stoma complication (ROR = 98.49), blood lactic acid abnormal (ROR = 74.88), coronavirus pneumonia (ROR = 69.41), and splenic embolism (ROR = 56.94).

### 3.8 Sensitivity analysis

The sensitivity analysis showed that all signals identified using the MCC remained statistically significant after FDR adjustment (*q* < 0.05). As illustrated in the UpSet plot (Supplementary Figure S5), the sets of signals before and after FDR correction exhibited complete overlap, with no changes in either the number or composition of detected signals. This high level of concordance indicates that the MCC framework inherently provides strong control over multiple comparisons and effectively filters out potential false-positive findings. Therefore, the sensitivity analysis offers robust support for our main results, confirming that the detected signals are not artifacts of multiple testing but are statistically stable and reliable.

## 4 Discussion

In this large real-world pharmacovigilance analysis of FAERS (2004–2024), we systematically mapped AE profiles of current FDA-approved ADCs onto their four key components: antibody subtype, linker type, payload category, and DAR class. We identified distinct, reproducible patterns of component-specific toxicity that extend current trial-based knowledge and recent mechanistic reviews of ADC safety (Chen et al., 2025; Cheng et al., 2025). MCC, a conservative four-tier screening criterion used in this study, together with the FDR sensitivity analysis confirming the robustness of the results, collectively ensures the validity of our findings.

A central finding was the enrichment of hepatobiliary injury, bone-marrow suppression, and infection-related signals among IgG4-based ADCs, whereas IgG1-based ADCs were more strongly associated with ocular toxicity and lymphoma-related events. The predominance of ocular and corneal toxicity among IgG1 ADCs is consistent with accumulating clinical data on BM and other microtubule-inhibitor ADCs, where microcystic keratopathy, punctate keratitis, and visual disturbances are frequent dose-limiting toxicities (Marquant et al., 2021). Several mechanistic hypotheses have been proposed: (i) off-target uptake of ADC or free payload by corneal epithelial cells *via* macropinocytosis from the tear film or limbal vasculature; (ii) microtubule disruption in basal epithelial and sub-basal nerve fibers leading to epithelial apoptosis and neurotrophic keratopathy; and (iii) Fc-mediated interactions with ocular surface immune cells that may amplify local injury (Zhao et al., 2018; Loberg et al., 2022; Mahalingaiah et al., 2019). In our dataset, the IgG1 group is enriched for microtubule-inhibitor-containing ADCs, which likely explains a large part of the ocular signal. The microtubule-inhibitor is MMAE or monomethyl auristatin F (MMAF). However, the near absence of comparable eye toxicity in the IgG4 group suggests that isotype-linked differences in tissue distribution, Fcγ receptor binding, and immune complex behavior may further modulate risk.

IgG1 has high affinity for activating Fcγ receptors and efficiently triggers antibody-dependent cellular cytotoxicity (ADCC), antibody-dependent cellular phagocytosis, and complement activation, making it the preferred backbone for cytotoxic and immune-effector therapeutic antibodies (van der Horst et al., 2020). This heightened effector capacity may favor local inflammatory injury in microvascular beds (including the ocular surface) when combined with potent cytotoxic payloads and repeated dosing. IgG4 has weak binding affinity for complement component C1q and Fcγ receptors, which limits its capacity to activate complement or mediate ADCC, and it undergoes Fab-arm exchange, reducing its ability to form large immune complexes. These features may partially protect against immune-mediated ocular surface injury but, at the same time, could alter pharmacokinetics and tissue distribution, potentially favoring prolonged systemic exposure and accumulation in hepatic, sinusoidal, or endothelial compartments (Wang et al., 2022).

The IgG4 group in this study (GO and IO) primarily carries DNA-damaging calicheamicin payloads, which are well known to cause hepatotoxicity, sinusoidal obstruction syndrome (SOS), veno-occlusive disease (VOD), and severe myelosuppression in both clinical trials and post-marketing cohorts. Our FAERS-derived signals for hepatobiliary disease, VOD, fibrin degradation product elevation, leukemic hepatic infiltration, sepsis, and neutropenic infection are therefore biologically plausible and closely aligned with prior observations.

Multiple clinical and real-world studies consistently show that ozogamicin-containing IgG4 ADCs (GO and IO) are strongly associated with hepatic SOS, VOD, and hepatotoxicity. Randomized trials and transplant cohorts report markedly elevated VOD rates, and GO product labeling also highlights VOD as a serious risk (Kantarjian et al., 2017; Sun et al., 2024; Kloehn et al., 2022). Our safety results are consistent with these findings. We observed very strong hepatobiliary signals (hepatobiliary disease: ROR 44.6; liver tenderness: ROR 54.5; VOD: ROR 295.7). Previous FAERS and observational studies likewise rank GO and IO among the drugs most disproportionately associated with SOS and VOD (Duan et al., 2025; Sun et al., 2025). Ozogamicin, a calicheamicin-derived cytotoxic payload, induces DNA double-strand breaks after internalization. Mechanistically, calicheamicin release within hepatic macrophages and sinusoidal endothelial cells can trigger endothelial injury and sinusoidal blockage, which may be further exacerbated by hematopoietic stem cell transplantation conditioning. Thus, the pronounced hepatotoxicity signals likely reflect payload-mediated endothelial damage rather than any intrinsic effect of the IgG4 backbone (Nguyen et al., 2023).

Taken together, the antibody subtype itself is unlikely to be a direct source of toxicity; rather, it may modulate payload-driven toxicities through its Fc-mediated functions, tissue distribution, and interactions with the conjugated payload.

Cleavable and non-cleavable linker ADCs showed clearly divergent toxicity spectra at the PT level. Cleavable linkers are engineered to release payload in response to pH, reducing conditions, or lysosomal proteases, enabling a strong bystander effect but at the cost of a higher risk of premature or systemic payload release (Takakura et al., 2024). A recent meta-analysis of clinical ADC trials showed that, after adjustment for DAR, cleavable linkers were independently associated with higher rates of grade ≥3 systemic toxicities and higher circulating free-payload concentrations, supporting the hypothesis that linker cleavability increases off-target exposure (Tang SC. et al., 2024). This provides a coherent explanation for the systemic, infection-prone, and ILD-like toxicity pattern we observed: higher free-payload exposure can aggravate bone-marrow suppression, endothelial injury, and immune dysfunction, thereby predisposing an individual to opportunistic infections, neutropenic colitis, and interstitial lung abnormalities flagged by KL-6 elevation.

For non-cleavable linkers, classical pharmacology would predict lower systemic off-target toxicity because these constructs are highly stable in plasma and require complete lysosomal degradation of the antibody to release the payload (Takakura et al., 2024). However, our data and accumulating clinical experience with BM, a B-cell maturation antigen (BCMA)-directed ADC with a non-cleavable maleimidocaproyl linker and MMAF payload, indicate that ocular surface toxicity can still be prominent and dose-limiting. Clinical series consistently describe microcyst-like epithelial changes, keratopathy, blurred vision, and dry eye as the dominant adverse events (Morè et al., 2023; Lonial et al., 2020). It is speculated that the mechanism may be ADC or its catabolites can reach the corneal surface via limbal vasculature or tear film, be internalized into corneal epithelial and limbal stem cells via macropinocytosis, and then induce local accumulation of a charged MMAF metabolite that cannot readily diffuse out, leading to focal apoptosis and microcystic keratopathy (Wahab et al., 2021). Our FAERS-based signal pattern for non-cleavable linker ADCs—markedly enriched for corneal epithelial lesions, ocular cysts, ocular surface disease, and night blindness—aligns well with these mechanistic observations.

Our results support the growing view that cleavable linkers should be used cautiously when systemic free-payload exposure is a major concern, whereas non-cleavable linkers may be preferable for targets with homogeneous antigen expression when minimizing systemic toxicity is critical. For cleavable linker ADCs, clinicians should prioritize systemic safety monitoring, including serial complete blood counts, liver function tests, and vigilance for respiratory symptoms and infection (e.g., neutropenic colitis and atypical pneumonia), especially in patients receiving DNA-damaging or highly myelosuppressive payloads. For non-cleavable linker ADCs, proactive ophthalmologic surveillance is essential. Baseline and periodic slit-lamp examinations, early recognition of keratopathy, and patient education regarding visual symptoms are critical to preventing irreversible ocular damage and avoiding premature drug discontinuation.

ADCs with DNA-damaging payloads showed a highly systemic and clinically severe toxicity pattern, characterized by hepatobiliary–vascular injury, ILD-like pulmonary toxicity, sepsis, bone marrow suppression, and coagulation abnormalities. This result is basically consistent with the known safety profile of calicheamicin- and topoisomerase I inhibitor-based ADCs such as GO, IO, and TD, which are strongly associated with hepatic sinusoidal obstruction/veno-occlusive disease, myelosuppression, and interstitial lung disease (Nguyen et al., 2023). SG similarly carries a high burden of severe neutropenia, neutropenic sepsis, and gastrointestinal toxicity, reinforcing that potent DNA-damage payloads drive a systemic, high-severity AE spectrum (Dacoregio et al., 2025). Mechanistically, DNA-damaging ADCs combine highly permeable linkers and membrane-permeable payloads, which can diffuse beyond target cells (“bystander effect”) and accumulate in non-malignant liver sinusoidal endothelial cells and alveolar epithelium. This may trigger sinusoidal obstruction, veno-occlusive liver disease, and ILD-like lung injury *via* endothelial damage, inflammation, and impaired tissue repair (Abuhelwa et al., 2022). Concomitant marrow suppression and neutropenia enhance susceptibility to bacterial translocation and sepsis, explaining the clustering of neutropenic colitis, perineal cellulitis, and septic events in our DNA-damaging payload group.

In contrast, microtubule inhibitor-based ADCs in this study (mainly MMAE-/MMAF-based) were dominated by ocular surface and corneal toxicity, treatment-related secondary lymphoma, and local hemorrhagic complications. This finding is consistent with the characteristic ocular toxicity observed clinically for ADCs bearing MMAE or MMAF payloads (Lu et al., 2023). The underlying mechanism has been discussed in detail in the section on the IgG1 subtype. Notably, although peripheral neuropathy accounted for a large number of AE reports within the microtubule-inhibitor payload group, its ROR was only moderate (13.61) and did not reach our predefined threshold for a strong signal. This pattern is consistent with prior clinical and pharmacovigilance evidence showing that ADCs carrying microtubule inhibitors can induce neurotoxic adverse events (Taoka et al., 2024; Tang L. et al., 2024). Mechanistically, microtubule inhibitors such as MMAE and MMAF disrupt microtubule polymerization and destabilize the axonal microtubule network, thereby impairing axonal transport and compromising the maintenance and repair of nerve terminals. These cellular disturbances ultimately lead to axonal degeneration, terminal retraction, and loss of sensory and conductive function—features well described in preclinical and clinical models of chemotherapy-induced peripheral neuropathy. In the context of ADCs, premature payload release from cleavable linkers, bystander diffusion, or uptake by perivascular endothelial cells may expose adjacent non-tumor neurons to cytotoxic metabolites. Transcytosis across the blood–nerve barrier may further deliver the payload into the peri-neural microenvironment, amplifying neurotoxic injury (Best et al., 2021).

The “others” payload group, dominated in current practice by immunotoxin-based constructs such as MP, showed a small but very high-ROR set of signals centered on endothelial injury, including capillary leak syndrome, hemolytic uremic syndrome, renal failure, and hypoxia. This finding is consistent with the boxed warnings and post-marketing experience for this class.

Our DAR-stratified analysis demonstrates that ADCs with DAR < 3 preferentially generate a hepatic, infectious, and hematologic toxicity pattern, whereas DAR 3–5 ADCs show a highly enriched profile of ocular surface and corneal injury and treatment-related secondary lymphoma events, and DAR > 5 ADCs exhibit the most heterogeneous multi-organ toxicity, spanning metabolic, hepatobiliary, pulmonary (ILD-like), hematologic–vascular, gastrointestinal, neurologic, and ocular domains. This gradient is broadly consistent with clinical and mechanistic data showing that higher DAR is associated with increased systemic toxicities and a narrower therapeutic index (Tang SC. et al., 2024). At present, many clinically successful ADCs fall within the DAR 3–5 group, which showed the most prominent clustering of ocular and corneal toxicities. Notably, five of the eight ADCs in this category employ microtubule-inhibitor payloads (MMAE/MMAF), consistent with the well-recognized ocular toxicity profile of this payload class (Lu et al., 2023). The toxicity profile of ADCs with a DAR < 3 closely mirrors that of the DNA-damaging payload class as all ADCs in this DAR category employ DNA-damaging agents. Their safety pattern aligns with the well-established classic toxicities of calicheamicin- or anthracycline-based drugs (Nguyen et al., 2023). ADCs with DAR > 5 show fewer but highly enriched strong signals across diverse organ systems, supporting the notion from preclinical studies that very high DARs increase hydrophobicity, hepatic uptake, rapid clearance, and systemic exposure to free payload, thereby amplifying off-target toxicities (Sun et al., 2017).

The DAR-stratified toxicity patterns indicate that DAR is not only a manufacturing parameter but also an important clinical variable that influences organ-specific toxicity. For patients with baseline hepatic impairment, high infection risk, or multimorbidity, monitoring strategies and treatment selection should be adapted according to DAR categories: ADCs with DAR < 3 require strengthened monitoring of hepatic function, bone marrow suppression, and infection markers; ADCs with DAR 3–5 warrant routine ophthalmologic assessment and prophylactic interventions; and ADCs with DAR > 5 should be used with caution in patients with poor overall performance status, with careful evaluation of the risk–benefit balance.

In summary, this real-world analysis shows that ADCs stratified by key components—including antibody subtype, linker type, payload class, and DAR category—exhibit clearly distinguishable toxicity profiles. However, these associations are strongly influenced by indication, co-medications, and interdependence among component types. For example, the ocular toxicity observed in IgG1-based ADCs likely does not arise from the antibody subtype itself but rather reflects the dominant use of MMAE/MMAF payloads and non-cleavable linkers, potentially reinforced by Fc-mediated tissue distribution. Thus, the component–toxicity patterns identified in this study should be interpreted as hypothesis-generating signals rather than evidence of causality. These findings provide a mechanistic framework for future ADC optimization and highlight the need for prospective, component-controlled studies to validate the observed associations.

### 4.1 Limitations

There are some limitations. First, the FAERS database is a spontaneous reporting system (https://open.fda.gov/data/faers/), which may be subject to incomplete or inaccurate information (Lin et al., 2024). Although we performed data cleaning to remove duplicate and withdrawn reports, some degree of error may still remain, potentially affecting the completeness and accuracy of our findings. Second, we employed MCC to minimize false positives, but some true signals may have been missed due to the inherent limitations of each approach (Shi et al., 2023). Third, differences in the structure, mechanisms, pharmacokinetics, and pharmacodynamics of various ADCs, along with the potential influence of combination therapies, may increase the risk of AEs. However, it is difficult to fully account for and adjust these factors in data analyses, which may introduce bias into the findings (Yang et al., 2024). Fourth, this study was limited to 15 ADCs approved by 2024. ADCs approved after the study period, along with newly emerging types of AEs, were not captured in this analysis. In addition, a substantial proportion of onset time data for adverse event reports was missing (63.41%), which greatly limits the reliability of these results. Finally, differences in medical practice across countries, regions, or healthcare institutions, along with individual patient differences, may also affect the generalizability of our findings.

## 5 Conclusion

This large real-world pharmacovigilance analysis identified 52,699 positive PT-level AE signals across 13 FDA-approved ADCs and demonstrated that key component types are strongly associated with distinct toxicity profiles. IgG1-based ADCs showed enriched ocular and corneal toxicity and treatment-related secondary lymphoma, whereas IgG4 constructs were characterized by hepatobiliary injury, vascular toxicity, bone marrow suppression, and severe infections. Cleavable linkers were associated with treatment-related secondary lymphoma and pulmonary- and infection-related toxicities, while non-cleavable linkers displayed a uniquely ocular-dominant pattern. DNA-damaging payloads produced severe systemic toxicities, whereas microtubule inhibitors primarily induced ocular injury and lymphoma-related events. DAR stratification further revealed ocular-dominant toxicity for DAR 3–5, hepatobiliary–infectious patterns for DAR < 3, and heterogeneous multi-organ toxicity for DAR > 5. These findings provide a component-based framework to inform ADC safety evaluation and guide future design.

## Data Availability

The original contributions presented in the study are included in the article/Supplementary Material; further inquiries can be directed to the corresponding author.

## Glossary

ADCs: Antibody–drug conjugates
AE: Adverse event
FDA: Food and Drug Administration
FAERS: Adverse Event Reporting System
ROR: Reporting odds ratio
PRR: Proportional reporting ratio
IC: Information component
EBGM: Empirical Bayesian geometric mean
MCC: Multi-method consensus criteria
PT: Preferred term
SOC: System organ class
DAR: Drug-to-antibody ratio
FDR: False discovery rate
GO: Gemtuzumab ozogamicin
BV: Brentuximab vedotin
TE: Ado-trastuzumab emtansine
IO: Inotuzumab ozogamicin
MP: Moxetumomab pasudotox-tdfk
PV: Polatuzumab vedotin
EV: Enfortumab vedotin
TD: Fam-trastuzumab deruxtecan-nxki
SG: Sacituzumab govitecan
BM: Belantamab mafodotin
CS: Cetuximab sarotalocan
LT: Loncastuximab tesirine
DV: Disitamab vedotin
TV: Tisotumab vedotin
MS: Mirvetuximab soravtansine
DD: Datopotamab deruxtecan
ILD: Interstitial lung disease
MMAE: Monomethyl auristatin E
MMAF: Monomethyl auristatin F
HSOS: Hepatic sinusoidal obstruction syndrome
VOD: Veno-occlusive disease
ADCC: Antibody-dependent cellular cytotoxicity
CDC: Complement-dependent cytotoxicity
BCMA: B-cell maturation antigen
OT: Onset time
Q1: Lower quartile
Q3: Upper quartile
