# Supplementary material for: A real-world study of adverse event profiles associated with the four key components of antibody–drug conjugates based on the FAERS database
Source: Front Pharmacol. 2026 Feb 13;17:1702195. doi: 10.3389/fphar.2026.1702195 (PMC12946140; doi:10.3389/fphar.2026.1702195)
Supplement: Supplementary file 1 [file Supplementaryfile1.docx]

Supplementary Material

**Supplementary Table 1. 14 FDA-approved antibody-drug conjugates**

| **Common name** | **Abbreviations** | **Trade names** | **Target antigens** | **Antibody type** | **Linkers** | **Payloads** | **Payload type** | **DAR** | **Approved indications** | **Approved date** | **Inclusion** | **Reason of exclusion** |
| --- | --- | --- | --- | --- | --- | --- | --- | --- | --- | --- | --- | --- |
| Gemtuzumab Ozogamicin | GO | Mylotarg | CD33 | IgG4 | Acid-labile hydrazone linker | calicheamicin | DNA-damaging agent | 2-3 | R/R CD33+ AML | 2000.05 2017.09 | Yes |  |
| Brentuximab Vedotin | BV | Adcetris | CD30 | IgG1 | Cleavable linker | MMAE | Microtubule inhibitor | 4 | R/R HL; sALCL | 2011.08 | Yes |  |
| Ado-trastuzumab Emtansine | TE | Kadcyla | HER2 | IgG1 | Non-cleavable thioether linker | DM1 | Microtubule inhibitor | 3.5 | HER2+ mBC; HER2+ eBC | 2013.02 | Yes |  |
| Inotuzumab Ozogamicin | IO | Besponsa | CD22 | 1gG4 | Acid-labile hydrazone linker | calicheamicin | DNA-damaging agent | 6 | R/R B‐cell precursor ALL | 2017.08 | Yes |  |
| Moxetumomab Pasudotox-tdfk | MP | Lumoxiti | CD22 | IgG1 | Protein fusion | PE38 | Others | 3-4 | R/R HCL | 2018.09 | Yes |  |
| Polatuzumab Vedotin | PV | Polivy | CD79b | IgG1 | Protease-cleavable linker (MC-VC-PABC) | MMAE | Microtubule inhibitor | 3.5 | R/R DLBCL | 2019.06 | Yes |  |
| Enfortumab Vedotin | EV | Padcev | Nectin-4 | IgG1 | Protease-cleavable linker (MC-VC-PABC) | MMAE | Microtubule inhibitor | 4 | Locally advanced or mUC | 2019.12 | Yes |  |
| Fam-trastuzumab deruxtecan-nxki | TD | Enhertu | HER2 | IgG1 | Cleavable tetrapeptide linker | Dxd | DNA-damaging agent | 8 | HER2+ mBC; HER2+ unresectable BC | 2019.12 | Yes |  |
| Sacituzumab Govitecan | SG | Trodelvy | TROP-2 | IgG1 | Cleavable CL2A linker | SN-38 | DNA-damaging agent | 7.6 | Locally advanced or mTNBC; | 2020.04 | Yes |  |
| Belantamab Mafodotin | BM | Blenrep | BCMA | IgG1 | Non-cleavable maleimidocaproyl linker | MMAF | Microtubule inhibitor | 4 | R/R multiple myeloma | 2020.08  2025.10 | Yes |  |
| Loncastuximab Tesirine | LT | Zynlonta | CD19 | IgG1 | Protease-cleavable linker | PBD | DNA-damaging agent | 2.3 | R/R DLBCL | 2021.04 | Yes |  |
| Tisotumab Vedotin | TV | Tivdak | Tissue factor | IgG1 | Protease-cleavable linker (MC-VC-PABC) | MMAE | Microtubule inhibitor | 4 | Recurrent or metastatic cervical cancer | 2021.09 | Yes |  |
| Mirvetuximab Soravtansine | MS | Elahere | FRα | IgG1 | Cleavable sulfo-SPDB linker | DM4 | Microtubule inhibitor | 3.3-5 | FRα+ platinum resistant epithelial ovarian, fallopian tube, or primary peritoneal cancer | 2022.11 | Yes |  |
| Datopotamab deruxtecan | DD | Datroway | TROP2 | IgG1 | protease cleavable linker | DXd | DNA-damaging agent | 4 | HR+/HER2 - BC | 2024.12.27 | NO | No positive signal data. |

Note: AML, acute myeloid leukemia; HL, Hodgkin lymphoma; sALCL, systemic anaplastic large cell lymphoma; MMAE, monomethyl auristatin E; HER2+, HER2-positive; mBC, metastatic breast cancer; DM1, emtansine; ALL, acute lymphoblastic leukemia; HCL, Hairy cell leukemia; PE38, pseudomonas exotoxin 38; DLBCL, diffuse large B-cell lymphoma; mUC, metastatic urothelial cancer; DXd, a derivative of exatecan; mTNBC, metastatic triple-negative breast cancer; PBD, pyrrolobenzodiazepine; DAR, drug–antibody ratio; DM4, ravtansine; R/R, relapsed or refractory; SN38, active metabolite of irinotecan; BCMA, B-cell maturation antigen; FRα, folate receptor alpha; HER2, human epidermal growth factor receptor 2; MMAF, monomethyl auristatin F; IRDye® 700DX, a photoactivatable dye; eBC, early breast cancer; NSCLC, non-small cell lung cancer; TNBC, triple-negative breast cancer; HR, hormone receptor;

**Supplementary Table 2.** 2×2 Table for ROR calculation by key component type of ADCs

|  | Event E reported | Event E not reported |
| --- | --- | --- |
| Key component type of interest | a | b |
| All other drugs | c | d |

Note: a = Number of FAERS reports where a primary suspect drug belongs to the IgG 1 type of interest (e.g. IgG1-based ADCs), and the adverse event E is reported. b = Number of FAERS reports where a suspect ADC belongs to the IgG1 type of interest, but event E is not reported. c = Number of FAERS reports where no suspect drug belongs to the IgG1 type of interest (e.g. only IgG4 ADCs or non-ADC drugs), but event E is reported. d = Number of FAERS reports where no suspect drug belongs to the IgG1 type of interest, and event E is not reported.

**Supplementary Table 3.** **Fo**ur signal detection methods

| **Method** | **Equation** | **Criteria** |
| --- | --- | --- |
| ROR | $\text{ROR=}\frac{\text{(a/c)}}{\text{(b/d)}}\text{=}\frac{\text{ad}}{\text{bc}}$  $\text{95\%Cl=}\text{e}^{\text{ln(ROR)}\text{±}\text{1.96}\sqrt{\text{(}\frac{\text{1}}{\text{a}}\text{+}\frac{\text{1}}{\text{b}}\text{+}\frac{\text{1}}{\text{c}}\text{+}\frac{\text{1}}{\text{d}}\text{)}}}$ | a ≥ 3 and the lower limit of the 95% confidence interval (ROR_025_) > 1 indicates the generation of a signal. |
| PRR | $\text{PRR=}\frac{\text{a/(a+b)}}{\text{c/(c+d)}}$  $\text{95\%Cl=}\text{e}^{\text{ln(ROR)}\text{±}\text{1.96}\sqrt{\text{(}\frac{\text{1}}{\text{a}}\text{+}\frac{\text{1}}{\text{a+b}}\text{+}\frac{\text{1}}{\text{c}}\text{+}\frac{\text{1}}{\text{c+d}}\text{)}}}$ | a ≥ 3 and the lower limit of the 95% confidence interval (PRR_025_) > 1 indicates the generation of a signal. |
| MGPS | $\text{EBGM=}\frac{\text{a(a+b+c+d)}}{\text{(a+c)(a+b)}}$  $\text{95\%Cl=}\text{e}^{\text{ln(EBMG)}\text{±}\text{1.96}\sqrt{\text{(}\frac{\text{1}}{\text{a}}\text{+}\frac{\text{1}}{\text{b}}\text{+}\frac{\text{1}}{\text{c}}\text{+}\frac{\text{1}}{\text{d}}\text{)}}}$ | EBGM_05_＞2 indicates the generation of a signal. |
| BCPNN | $\text{IC=log2}\frac{\text{p(x,y)}}{\text{p(x)p(y)}}\text{=log2}\frac{\text{a(a+b+c+d)}}{\text{(a+b)(a+c)}}$  $\text{E(IC)=log2}\frac{\text{(a+}\text{γ}\text{11)(a+b+c+d+}\text{α}\text{)(a+b+c+d+}\text{β}\text{)}}{\text{(a+b+c+d+}\text{γ}\text{)(a+b+}\text{α}\text{1)(a+c+}\text{β}\text{1)}}$  $\text{V(IC)=}\frac{\text{1}}{\text{(ln2)}^{\text{2}}}\left\{ \left[ \frac{\text{(a+b+c+d)-a+}\text{γ}\text{-}\text{γ}\text{11}}{\text{(a+}\text{γ}\text{11)(1+a+b+c+d+}\text{γ}} \right]\text{+}\left[ \frac{\text{(a+b+c+d)-(a+b)+}\text{α}\text{-}\text{α}\text{1}}{\text{(a+b+}\text{α}\text{1)(1+a+b+c+d+}\text{α}} \right]\text{+}\left[ \frac{\text{(a+b+c+d)-(a+c)+}\text{β}\text{-}\text{β}\text{1}}{\text{(a+c+}\text{β}\text{1)(1+a+b+c+d+}\text{β}\text{)}} \right] \right\}$  $\text{γ}\text{=γ11}\frac{\text{(a+b+c+d+}\text{α}\text{)(a+b+c+d+}\text{β}\text{)}}{\text{(a+b+}\text{α}\text{1)(a+c+}\text{β}\text{1)}}$  $\text{IC-2SD=E(IC)-2}\sqrt{\text{V(IC)}}$  $\text{其中}\text{α1=β1=1;α=β=2;γ11=1}$ | The lower limit of the confidence interval (IC-2SD)＞0 indicates the generation of a signal. |

Notes: Equation: a, number of reports containing both the target drug and the target adverse drug reaction; b, number of reports containing other adverse drug reactions of the target drug; c, number of reports containing the target adverse drug reaction of other drugs; d, number of reports containing other drugs and other adverse drug reactions. Abbreviations: 95% CI, 95% confidence interval; a, the number of reports; IC, information component; IC025, the lower limit of the 95% CI of the IC; E (IC), the IC expectations; V (IC), the variance of IC; EBGM, empirical Bayesian geometric mean; EBGM05, empirical Bayesian geometric mean lower 95% CI for the posterior distribution.

**Supplementary Table 4.** General characteristics of AE reports with 13 ADCs

| **Characteristics** | | **GO** | **BV** | **TE** | **IO** | **MP** | **PV** | **EV** | **TD** | **SG** | **BM** | **LT** | **TV** | **MS** |
| --- | --- | --- | --- | --- | --- | --- | --- | --- | --- | --- | --- | --- | --- | --- |
| Number of people | | 1921 | 6990 | 4854 | 1034 | 58 | 2291 | 3126 | 7497 | 3798 | 2122 | 215 | 500 | 752 |
| Number of events | | 8795 | 22636 | 13495 | 2797 | 153 | 6171 | 9784 | 19226 | 11159 | 7432 | 416 | 1083 | 1417 |
| SEX | Female | 714 (37.2) | 2224 (31.8) | 4102 (84.5) | 363 (35.1) | 10 (17.2) | 798 (34.8) | 699 (22.4) | 5775 (77.0) | 3306 (87.0) | 415 (19.6) | 61 (28.4) | 363 (72.6) | 476 (63.3) |
|  | Male | 881 (45.9) | 2948 (42.2) | 138 (2.8) | 469 (45.4) | 40 (69.0) | 1098 (47.9) | 2302 (73.6) | 752 (10.0) | 299 (7.9) | 512 (24.1) | 105 (48.8) | 13 (2.6) | 3 (0.4) |
|  | Missing | 326 (17.0) | 1818 (26.0) | 614 (12.6) | 202 (19.5) | 8 (13.8) | 395 (17.2) | 125 (4.0) | 970 (12.9) | 193 (5.1) | 1195 (56.3) | 49 (22.8) | 124 (24.8) | 273 (36.3) |
| AGE | ＜18 | 86 (4.5) | 447 (6.4) | 75 (1.5) | 136 (13.2) | - | 220 (9.6) | 428 (13.7) | 186 (2.5) | 10 (0.3) | - | - | 2 (0.4) | - |
|  | 18～64 | 715 (37.2) | 2376 (34.0) | 2012 (41.5) | 474 (45.8) | 8 (13.8) | 458 (20.0) | 412 (13.2) | 1785 (23.8) | 1405 (37.0) | 219 (10.3) | 44 (20.5) | 110 (22.0) | 62 (8.2) |
|  | 65～85 | 463 (24.1) | 1069 (15.3) | 655 (13.5) | 163 (15.8) | 22 (37.9) | 927 (40.5) | 1330 (42.5) | 1109 (14.8) | 511 (13.5) | 357 (16.8) | 69 (32.1) | 34 (6.8) | 70 (9.3) |
|  | ＞85 | 9 (0.5) | 62 (0.9) | 25 (0.5) | 3 (0.3) | - | 68 (3.0) | 69 (2.2) | 24 (0.3) | 10 (0.3) | 14 (0.7) | 7 (3.3) | - | 1 (0.1) |
|  | Missing | 648 (33.7) | 3036 (43.4) | 2087 (43.0) | 258 (25.0) | 28 (48.3) | 618 (27.0) | 887 (28.4) | 4393 (58.6) | 1862 (49.0) | 1532 (72.2) | 95 (44.2) | 354 (70.8) | 619 (82.3) |
| Outcomes | Death | 259 (13.5) | 1497 (21.4) | 754 (15.5) | 446 (43.1) | 11 (19.0) | 671 (29.3) | 602 (19.3) | 1827 (24.4) | 954 (25.1) | 814 (38.4) | 53 (24.7) | 32 (6.4) | 48 (6.4) |
|  | Disability | 2 (0.1) | 87 (1.2) | 42 (0.9) | 6 (0.6) | - | 12 (0.5) | 14 (0.4) | 28 (0.4) | 77 (2.0) | 15 (0.7) | - | 6 (1.2) | 3 (0.4) |
|  | Hospitalization | 176 (9.2) | 2142 (30.6) | 1129 (23.3) | 219 (21.2) | 17 (29.3) | 528 (23.0) | 778 (24.9) | 1296 (17.3) | 777 (20.5) | 368 (17.3) | 58 (27.0) | 86 (17.2) | 70 (9.3) |
|  | Life-Threatening | 122 (6.4) | 318 (4.5) | 123 (2.5) | 38 (3.7) | 2 (3.4) | 83 (3.6) | 96 (3.1) | 197 (2.6) | 127 (3.3) | 32 (1.5) | 1 (0.5) | 4 (0.8) | 6 (0.8) |
|  | Other | 1362 (70.9) | 2946 (42.1) | 2806 (57.8) | 325 (31.4) | 28 (48.3) | 997 (43.5) | 1636 (52.3) | 4149 (55.3) | 1863 (49.1) | 893 (42.1) | 103 (47.9) | 372 (74.4) | 625 (83.1) |
| Reporter  type | Health professional | 1253 (65.2) | 4854 (69.4) | 3497 (72.0) | 822 (79.5) | 41 (70.7) | 2071 (90.4) | 2159 (69.1) | 6124 (81.7) | 3317 (87.3) | 1670 (78.7) | 63 (29.3) | 437 (87.4) | 540 (69.8) |
|  | Consumer | 42 (2.2) | 1296 (18.5) | 1042 (21.5) | 151 (14.6) | 6 (10.3) | 210 (9.2) | 949 (30.4) | 1363 (18.2) | 474 (12.5) | 450 (21.2) | 29 (13.5) | 62 (12.4) | 241 (32.0) |
|  | Missing | 626 (32.6) | 840 (12.0) | 315 (6.5) | 61 (5.9) | 11 (19.0) | 10 (0.4) | 16 (0.5) | 5 (0.1) | 7 (0.2) | 2 (0.1) | 123 (57.2) | 1 (0.2) | 6 (0.8) |
| Country | United States | 663 (34.5) | 2163 (30.9) | 1783 (36.7) | 357 (34.5) | 47 (81.0) | 273 (11.9) | 1226 (39.2) | 3305 (44.1) | 1248 (32.9) | 1281 (60.4) | 137 (63.7) | 441 (88.2) | 723 (96.1) |
|  | Japan | 231 (12.0) | 927 (13.3) | 427 (8.8) | 157 (15.2) | - | 918 (40.1) | 1220 (39.0) | 847 (11.3) | 11 (0.3) | 5 (0.2) | - | 2 (0.4) | - |
|  | France | 210 (10.9) | 525 (7.5) | 234 (4.8) | 55 (5.3) | - | 32 (1.4) | 150 (4.8) | 606 (8.1) | 541 (14.2) | 246 (11.6) | 3 (1.4) | 7 (1.4) | 1 (0.1) |
|  | Canada | 21 (1.1) | 84 (1.2) | 150 (3.1) | 33 (3.2) | 2 (3.4) | 25 (1.1) | 41 (1.3) | 1351 (18.0) | 375 (9.9) | 54 (2.5) | - | 2 (0.4) | - |
|  | China | 1 (0.1) | 226 (3.2) | 280 (5.8) | 19 (1.9) | - | 149 (6.5) | 45 (1.5) | 234 (3.1) | 164 (4.3) | 34 (1.6) | 38 (17.7) | 3 (0.6) | 6 (0.8) |
|  | Others | 795 (41.4) | 3065 (43.8) | 1980 (40.8) | 413 (39.9) | 9 (15.5) | 894 (39.0) | 444 (14.2) | 1154 (15.4) | 1459 (38.4) | 502 (23.7) | 37 (17.2) | 45 (9.0) | 22 (2.9) |

**Supplementary Table 5.** Top 20 adverse event signals related to 13 ADCs

| **GO** | **BV** | **TE** | **IO** | **MP** | **PV** | **EV** | **TD** | **SG** | **BM** | **LT** | **TV** | **MS** |
| --- | --- | --- | --- | --- | --- | --- | --- | --- | --- | --- | --- | --- |
| Febrile neutropenia (332) | Off label use (798) | Disease progression (306) | Death (204) | Capillary leak syndrome (13) | Disease progression (266) | Rash (420) | Nausea (935) | Disease progression (1166) | Keratopathy (771) | Death (25) | Neuropathy peripheral (36) | Vision blurred (63) |
| Pyrexia (209) | Pyrexia (476) | Platelet count decreased (266) | Neoplasm progression (134) | Haemolytic uraemic syndrome (12) | Death (243) | Neuropathy peripheral (314) | Death (871) | Death (563) | Visual acuity reduced (708) | Rash (13) | Dry eye (33) | Diarrhoea (54) |
| Sepsis (197) | Neuropathy peripheral (440) | Thrombocytopenia (240) | Venoocclusive liver disease (98) | Weight increased (7) | Neutrophil count decreased (162) | Malignant neoplasm progression (308) | Interstitial lung disease (777) | Diarrhoea (449) | Death (498) | Pleural effusion (12) | Rash (26) | Neuropathy peripheral (52) |
| Platelet count decreased (187) | Hodgkin's disease (430) | Pyrexia (185) | Pyrexia (64) | Hypoxia (4) | Platelet count decreased (152) | Decreased appetite (208) | Off label use* (719) | Neutropenia (406) | Dry eye (359) | Photosensitivity reaction (10) | Product temperature excursion issue* (22) | Pneumonitis (46) |
| White blood cell count decreased (154) | Febrile neutropenia (420) | Neuropathy peripheral (176) | Platelet count decreased (62) | Blood creatinine increased (4) | Anaemia (137) | Pruritus (180) | Disease progression (621) | Inappropriate schedule of product administration* (302) | Plasma cell myeloma (337) | Oedema (10) | Eye disorder (18) | Abdominal pain (45) |
| Venoocclusive liver disease (150) | Neutropenia (341) | Epistaxis (152) | Febrile neutropenia (58) | Hypervolaemia (3) | Febrile neutropenia (128) | Alopecia (165) | Fatigue (617) | Febrile neutropenia (186) | Night blindness (317) | Platelet count decreased (9) | Conjunctivitis (17) | Cataract (40) |
| Aspartate aminotransferase increased (141) | Disease progression (215) | Metastases to central nervous system (151) | Venoocclusive disease (47) | Platelet count decreased (3) | Covid-19* (121) | Taste disorder (145) | Vomiting (400) | Asthenia (175) | Photophobia (182) | Pneumonia (9) | Anaemia* (17) | Infusion related reaction (38) |
| Chills (134) | Anaemia (213) | Myelosuppression* (142) | White blood cell count decreased (42) | Renal failure (3) | White blood cell count decreased (120) | Hyperglycaemia (143) | Pneumonitis (334) | Weight fluctuation (164) | Foreign body sensation in eyes (151) | Disease progression (8) | Alopecia (15) | Disease progression (38) |
| Pneumonia (134) | White blood cell count decreased (199) | No adverse event (142) | Thrombocytopenia (41) |  | Pyrexia (115) | Pyrexia (130) | No adverse event* (292) | Alopecia (150) | Vision blurred (126) | Gamma-glutamyltransferase increased (7) | Ocular toxicity (13) | Keratitis (32) |
| Infusion related reaction (119) | Sepsis (191) | Aspartate aminotransferase increased (103) | Blood bilirubin increased (35) |  | Neutropenia (105) | Skin disorder (126) | Decreased appetite (262) | Weight decreased (134) | Ocular toxicity (98) | Pericardial effusion (6) | Malignant neoplasm progression (13) | Dry eye (29) |
| Disease progression (117) | Thrombocytopenia (169) | Alanine aminotransferase increased (81) | Sepsis (35) |  | Lymphocyte count decreased (78) | Inappropriate schedule of product administration* (111) | Alopecia (252) | Neutrophil count decreased (113) | Punctate keratitis (92) | Thrombocytopenia (6) | Vaginal haemorrhage* (12) | Visual impairment (25) |
| Thrombocytopenia (110) | Myelosuppression (156) | Chills (69) | Neutropenia (30) |  | Myelosuppression (72) | Stevens-johnson syndrome (105) | Intentional product use issue* (219) | Anaemia (107) | Visual impairment (90) | Oedema peripheral (6) | Epistaxis (12) | Keratopathy (22) |
| Blood bilirubin increased (105) | Neutrophil count decreased (156) | Interstitial lung disease (65) | Ascites (27) |  | Neuropathy peripheral (67) | Myelosuppression (96) | Neutropenia (208) | Thrombocytopenia (85) | Eye disorder (74) | Neutropenia (6) | Muscular weakness (11) | Carbohydrate antigen 125 increased* (18) |
| Alanine aminotransferase increased (100) | Platelet count decreased (138) | Blood bilirubin increased (62) | Graft versus host disease* (25) |  | Thrombocytopenia (64) | Neutropenia (95) | Anaemia (191) | Sepsis* (82) | Corneal epithelial microcysts (73) | Anaemia (6) | Keratitis (10) | Abdominal pain upper (18) |
| Neutropenia (100) | General physical health deterioration* (133) | Pneumonitis (57) | Cytokine release syndrome* (24) |  | Cytopenia (56) | Anaemia (91) | Malignant neoplasm progression (172) | White blood cell count decreased (75) | Thrombocytopenia (71) | Diffuse large b-cell lymphoma (4) | Hospice care (9) | Ocular toxicity (17) |
| Haemoglobin decreased (95) | Polyneuropathy (131) | Hepatic cirrhosis (55) | Neutrophil count decreased (23) |  | Sepsis (56) | Toxic epidermal necrolysis (72) | Toxicity to various agents (165) | Heart rate increased (70) | Keratitis (67) | Hepatotoxicity (4) | Neoplasm progression (9) | Thrombocytopenia (13) |
| Hypotension (90) | Pancytopenia (122) | Hepatic function abnormal (54) | Multiple organ dysfunction syndrome (23) |  | No adverse event (53) | Febrile neutropenia (71) | Myelosuppression* (147) | Colitis (68) | Blindness (64) | Neutrophil count decreased (4) | Ocular hyperaemia (9) | Eye irritation (11) |
| Weight increased (90) | Anaplastic large cell lymphoma t- and null-cell types* (108) | Ejection fraction decreased (52) | Neoplasm recurrence (21) |  | Diffuse large b-cell lymphoma (50) | Hypoaesthesia (66) | Neutrophil count decreased (140) | Metastases to central nervous system* (62) | Corneal disorder (62) | Blister (4) | Cataract (9) | Eye disorder (9) |
| Pancytopenia (86) | Pneumocystis jirovecii pneumonia (101) | Hepatic enzyme increased (52) | Aspartate aminotransferase increased (20) |  | Infection (48) | Disease progression (63) | Platelet count decreased (126) | Neutropenic colitis (58) | Cataract (57) | Drug intolerance* (4) | Febrile neutropenia* (9) | Eye pain (9) |
| Blood alkaline phosphatase increased (85) | Haemoglobin decreased (101) | Liver disorder (50) | Infection (20) |  | Cytomegalovirus infection (45) | Hepatic function abnormal (60) | Thrombocytopenia (124) | Myelosuppression (58) | Hospitalisation (57) | Rash vesicular (3) | Visual impairment (9) | Photophobia (8) |

Notes: *AEs that are not mentioned in the drug label.

**Supplementary Table 6.** Top 20 most frequently reported adverse events by signal intensity for 13 ADCs

| **GO** | **BV** | **TE** | **IO** | **MP** | **PV** | **EV** | **TD** | **SG** | **BM** | **LT** | **TV** | **MS** |
| --- | --- | --- | --- | --- | --- | --- | --- | --- | --- | --- | --- | --- |
| Perineal cellulitis* (403.34) | Anaplastic large cell lymphoma t- and null-cell types refractory (1472.44) | Postmastectomy lymphoedema syndrome (650.12) | Venoocclusive liver disease (474.33) | Capillary leak syndrome (2956.09) | Diffuse large b-cell lymphoma recurrent (231.67) | Toxic erythema of chemotherapy (420.97) | Bartholin's cyst removal* (2889.73) | Eastern cooperative oncology group performance status abnormal (510.79) | Corneal epithelial microcysts(13442.14) | Rash vesicular (97.46) | Symblepharon (1125.41) | Eye abrasion (4911.97) |
| Fibrin degradation products increased* (340.20) | Hodgkin's disease refractory (1248.70) | Hepatopulmonary syndrome (560.10) | Venoocclusive disease (419.43) | Haemolytic uraemic syndrome (2115.09) | Diffuse large b-cell lymphoma refractory (193.17) | Kl-6 increased*(324.74) | Gastroenteritis listeria (1926.69) | Triple negative breast cancer (241.72) | Keratopathy (10646.87) | Photosensitivity reaction (91.41) | Ocular toxicity (900.10) | Corneal epithelial microcysts(1062.05) |
| Venoocclusive liver disease (229.45) | Hodgkin's disease recurrent (916.46) | Paravenous drug administration (484.44) | Neoplasm recurrence(137.53) | Hypervolaemia (61.06) | Lymphoma transformation* (170.01) | Leukoderma* (127.65) | Interstitial lung abnormality(279.64) | Neutropenic colitis (172.87) | Corneal cyst (5631.66) | Intercepted medication error* (90.47) | Punctate keratitis (476.25) | Ocular toxicity (904.42) |
| Serratia sepsis (199.55) | Anaplastic large cell lymphoma t- and null-cell types (799.62) | Spider naevus* (446.96) | Graft versus host disease in liver* (126.31) | Hypoxia (47.35) | Cytomegalovirus enterocolitis(143.60) | Sjs-ten overlap (88.59) | Kl-6 increased*(187.15) | Cholinergic syndrome (136.20) | Corneal toxicity (2107.60) | Diffuse large b-cell lymphoma(84.80) | Product temperature excursion issue* (376.0) | Keratopathy (647.64) |
| Refractoriness to platelet transfusion* (189.57) | Cutaneous t-cell lymphoma refractory (516.67) | Subcapsular hepatic haematoma* (425.94) | Neoplasm progression(84.12) | Blood creatinine increased*(24.58) | Cytomegalovirus test positive (94.28) | Transitional cell carcinoma(79.43) | Urinary tract stoma complication (156.20) | Weight fluctuation(94.13) | Night blindness*(2055.88) | Sunburn (58.39) | Peripheral motor neuropathy(204.21) | Keratitis (495.46) |
| Blast cell count increased (97.44) | Cutaneous t-cell lymphoma recurrent (426.96) | Radiation necrosis (311.42) | Graft versus host disease* (75.53) | Weight increased (13.42) | Metastatic lymphoma(92.64) | Symmetrical drug-related intertriginous and flexural exanthema(78.81) | Blood lactic acid abnormal (118.75) | Disease progression(62.13) | Ocular toxicity (1116.37) | Pneumonia bacterial (52.16) | Keratitis (198.17) | Punctate keratitis (310.89) |
| Venoocclusive disease (94.69) | Peripheral t-cell lymphoma unspecified(399.59) | Nodular regenerative hyperplasia(254.97) | Enterocolitis infectious (67.72) | Platelet count decreased (11.41) | Cytomegalovirus hepatitis* (84.18) | Cutaneous symptom (72.45) | Coronavirus pneumonia(110.08) | Vascular device occlusion (60.24) | Vital dye staining cornea present* (1038.97) | Gamma-glutamyltransferase increased (45.69) | Ulcerative keratitis (166.93) | Corneal epithelium defect (238.64) |
| Neutrophil percentage decreased (81.39) | Enteropathy-associated t-cell lymphoma(370.51) | Non-cirrhotic portal hypertension (202.14) | Graft versus host disease in skin* (60.51) | Renal failure (8.78) | Tumour associated fever (78.56) | Mechanical ileus* (70.58) | Splenic embolism*(90.30) | Discontinued product administered(59.52) | Punctate keratitis (1033.18) | Pericardial effusion (39.92) | Corneal disorder (102.43) | Carbohydrate antigen 125 increased*(217.68) |
| Liver tenderness (72.35) | Angioimmunoblastic t-cell lymphoma(365.28) | Intracranial tumour haemorrhage(86.58) | Bone marrow transplant (59.30) |  | Diffuse large b-cell lymphoma(71.86) | Skin toxicity (69.49) | Pseudocirrhosis (89.62) | Neutrophil count abnormal (57.27) | Corneal defect (966.15) | Pleural effusion (29.55) | Intercepted medication error* (92.79) | Corneal deposits (177.54) |
| Blast cells present (71.50) | Hodgkin's disease (349.07) | Metastases to spinal cord (84.60) | Splenic infarction*(50.72) |  | Tumour invasion* (62.99) | Tumour associated fever (66.25) | Radiation necrosis* (84.84) | Breast haemorrhage* (48.66) | Ocular cyst (854.81) | Oedema (28.04) | Conjunctivitis (54.99) | Peripheral motor neuropathy(93.26) |
| Intravascular haemolysis (62.09) | Peripheral t-cell lymphoma unspecified recurrent (250.44) | Telangiectasia* (76.69) | Hepatorenal syndrome (37.81) |  | Follicular lymphoma(62.35) | Peripheral motor neuropathy(63.53) | Eye haematoma(80.28) | Febrile bone marrow aplasia (42.83) | Ocular surface disease (617.87) | Hepatotoxicity* (27.61) | Hospice care (54.71) | Pneumonitis(79.64) |
| Neutropenic infection (56.39) | Axonal and demyelinating polyneuropathy* (142.27) | Portal hypertension(63.81) | Tumour lysis syndrome (37.09) |  | Abdominal lymphadenopathy* (60.23) | Taste disorder (54.44) | Tumour marker decreased (75.06) | Dose calculation error (40.33) | Ophthalmological examination abnormal (531.31) | Product preparation error* (23.11) | Dry eye(44.49) | Ovarian cancer (31.99) |
| Enterobacter sepsis (55.92) | Adult t-cell lymphoma/leukaemia(136.13) | Her2 positive breast cancer (63.69) | Cytokine release syndrome*(36.75) |  | Cytopenia(53.23) | Epidermal necrosis (52.86) | Breast haemorrhage* (57.04) | Enterocolitis infectious (39.76) | Corneal epithelium defect (499.38) | Neutrophil count decreased (15.08) | Eye discharge (41.98) | Cataract (31.15) |
| Neutropenic colitis (55.12) | Primary mediastinal large b-cell lymphoma(107.88) | Central nervous system necrosis (63.35) | Capillary leak syndrome*(34.23) |  | Cytomegalovirus infection reactivation(50.60) | Infusion site vesicles (52.84) | Interstitial lung disease (55.83) | Vein collapse (37.67) | Corneal thickening(400.64) | Platelet count decreased (12.70) | Eye disorder (32.26) | Extravasation(31.06) |
| Blood culture positive (52.42) | Cutaneous t-cell lymphoma(96.22) | Focal nodular hyperplasia(59.69) | Graft versus host disease in gastrointestinal tract* (29.74) |  | Hepatobiliary disease (50.04) | Ocular toxicity (52.61) | Lung opacity (54.0) | Infusion related hypersensitivity reaction (37.54) | Cornea verticillata(379.98) | Hepatic function abnormal (12.33) | Ocular discomfort(29.60) | Dry eye(29.57) |
| Lower respiratory tract infection fungal* (50.42) | Peripheral motor neuropathy(84.28) | Metastases to central nervous system (57.78) | Pneumonia fungal (29.72) |  | Tumour lysis syndrome (45.83) | Administration site extravasation(42.14) | Abdominal cavity drainage (50.70) | Metastases to meninges*(36.79) | Persistent corneal epithelial defect (373.98) | Blister (10.94) | Neuropathy peripheral(22.80) | Infusion related reaction (27.01) |
| Enterococcal bacteraemia* (49.06) | Peroneal nerve injury* (81.80) | Mean platelet volume decreased (51.42) | Cytopenia(29.11) |  | Heat illness* (44.74) | Cancer pain (36.27) | Metastases to meninges (46.15) | Neutropenic sepsis* (30.95) | Corneal dystrophy (241.54) | Disease progression(10.31) | Eye inflammation(19.32) | Neuropathy peripheral (25.27) |
| Central nervous system haemorrhage (44.71) | Glutamate dehydrogenase increased (79.16) | Breast cellulitis* (44.75) | Blood bilirubin increased (28.14) |  | Lymphocyte count decreased (44.09) | Dermatitis bullous (33.78) | Pneumonitis(42.48) | Cell death (30.78) | Corneal disorder (239.31) | Interstitial lung disease* (9.46) | Vaginal haemorrhage* (15.59) | Vision blurred (21.23) |
| Clostridium colitis (44.02) | Product temperature excursion issue* (77.41) | Breast haemorrhage* (40.24) | Second primary malignancy(25.76) |  | Neutrophil count decreased (42.05) | Toxic epidermal necrolysis(30.88) | Mitral valve replacement(40.03) | Xerophthalmia* (30.55) | Foreign body sensation in eyes (218.66) | Leukopenia(8.97) | Neoplasm progression(13.96) | Photophobia(19.26) |
| Biopsy bone marrow abnormal (42.50) | Beta 2 microglobulin increased*(64.59) | Gastric varices (39.50) | Portal hypertension* (21.64) |  | Gastrointestinal perforation(41.27) | Chronic inflammatory demyelinating polyradiculoneuropathy* (30.71) | Lymphangiosis carcinomatosa* (39.77) | Congenital aplasia (29.87) | Keratitis (197.73) | Thrombocytopenia (8.11) | Ocular hyperaemia(11.68) | Brain fog* (16.53) |

Notes: *AEs that are not mentioned in the drug label.


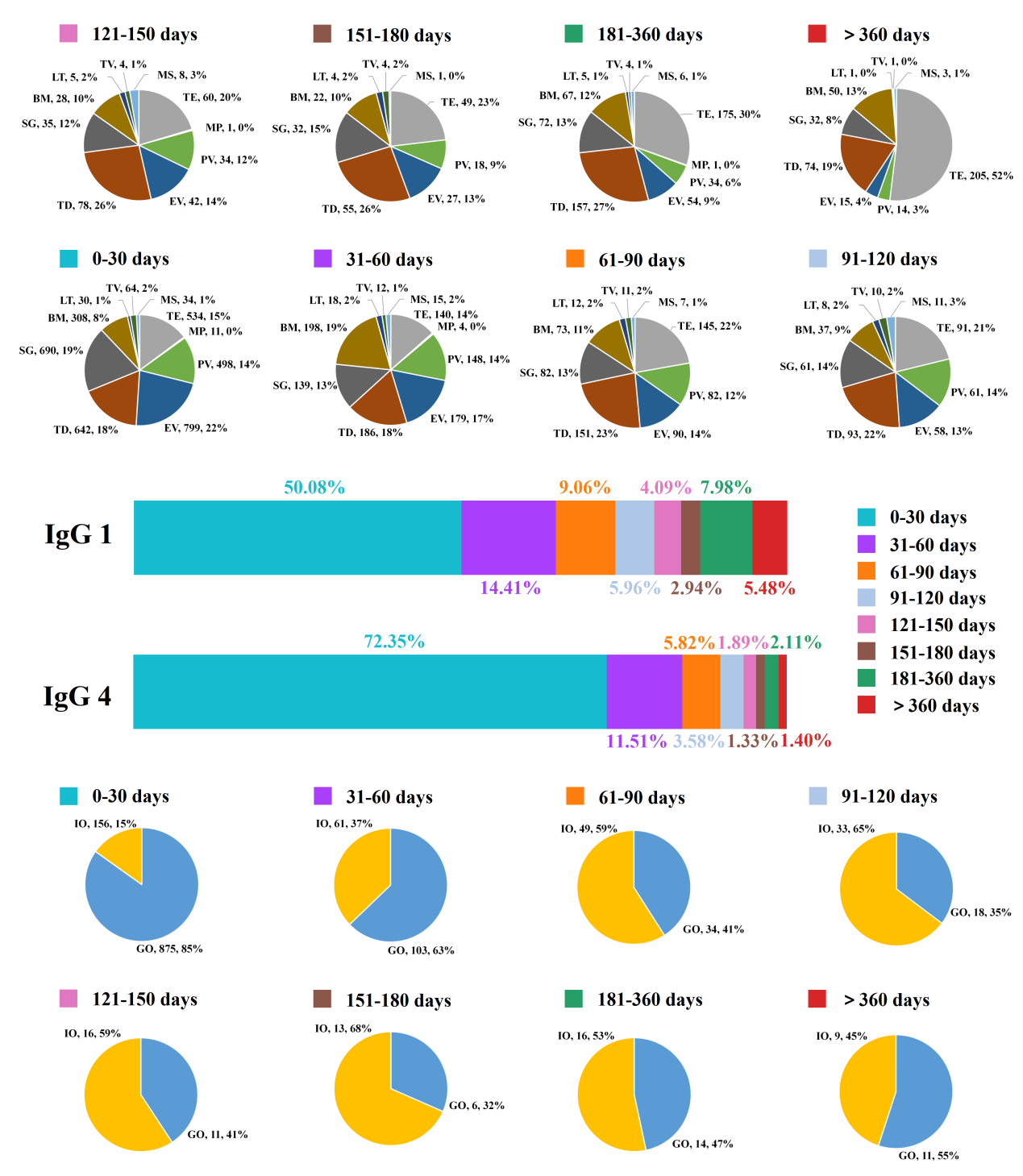


**Supplementary Figure 1.** Onset time of AEs related to IgG1 and IgG4 antibodies


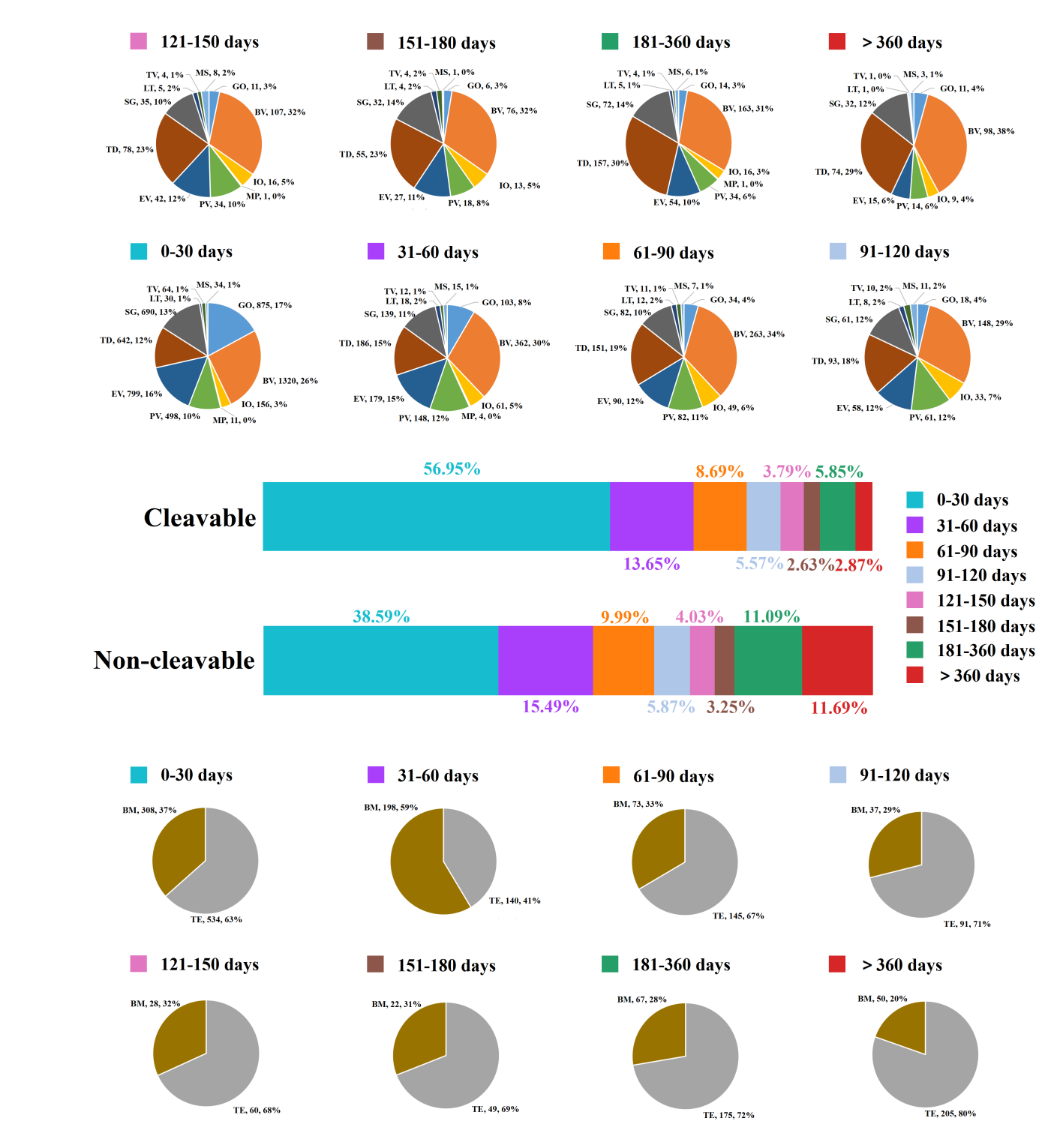


**Supplementary Figure 2.** Onset time of AEs related to cleavable and non-cleavable Linkers


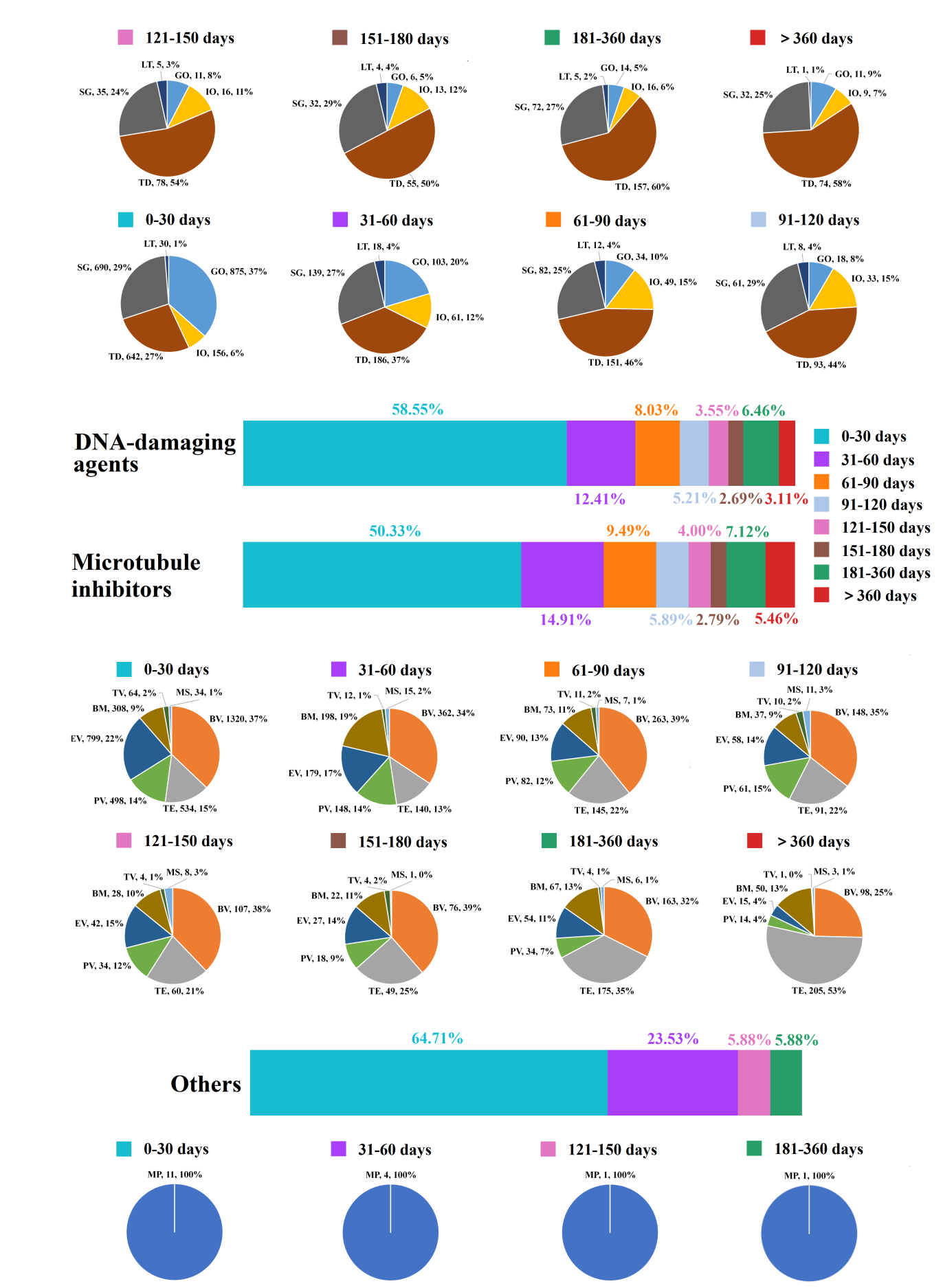


**Supplementary Figure 3.** Onset Time of AEs related to payload types


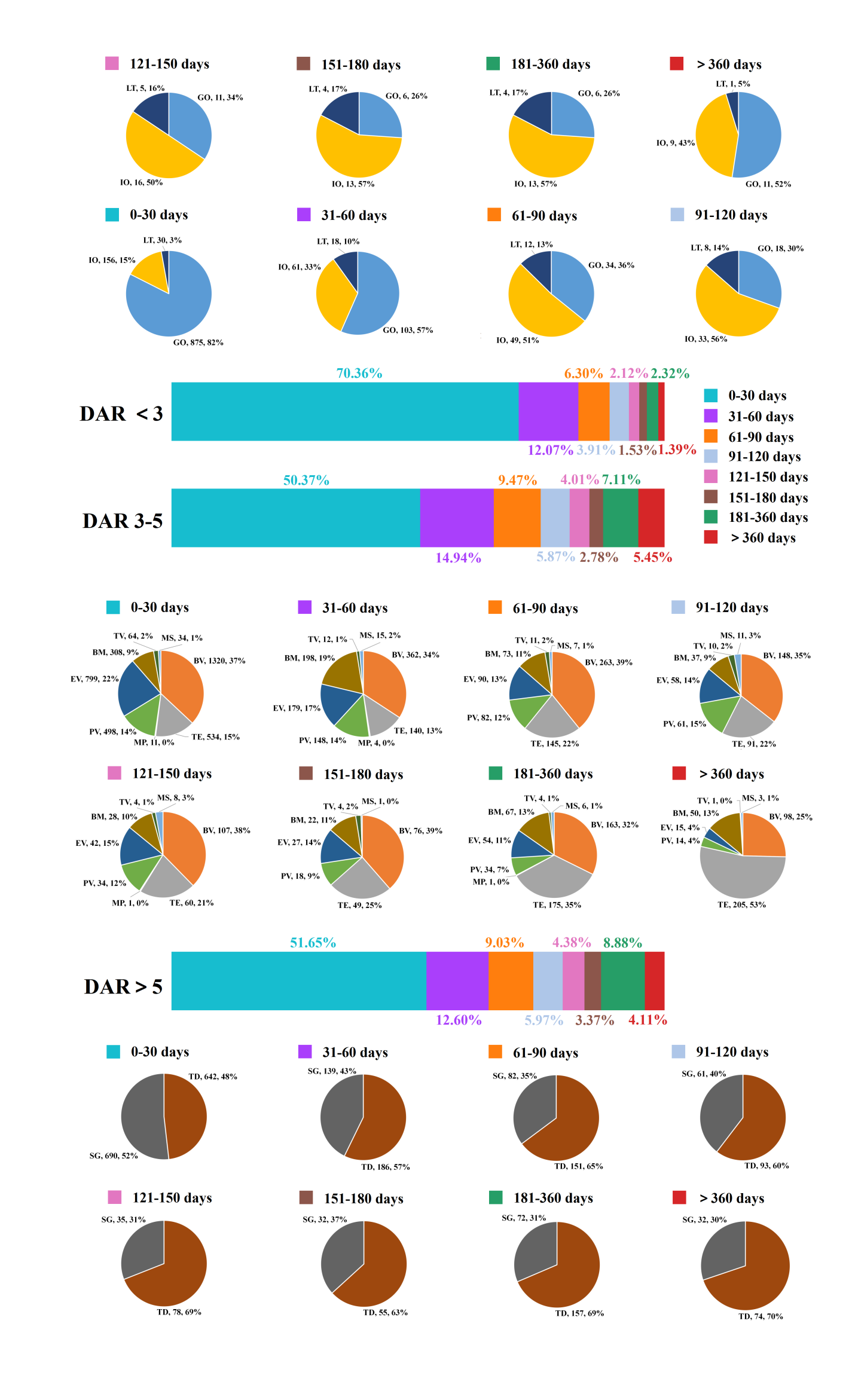


**Supplementary Figure 4.** Onset Time of AEs related to DAR Values


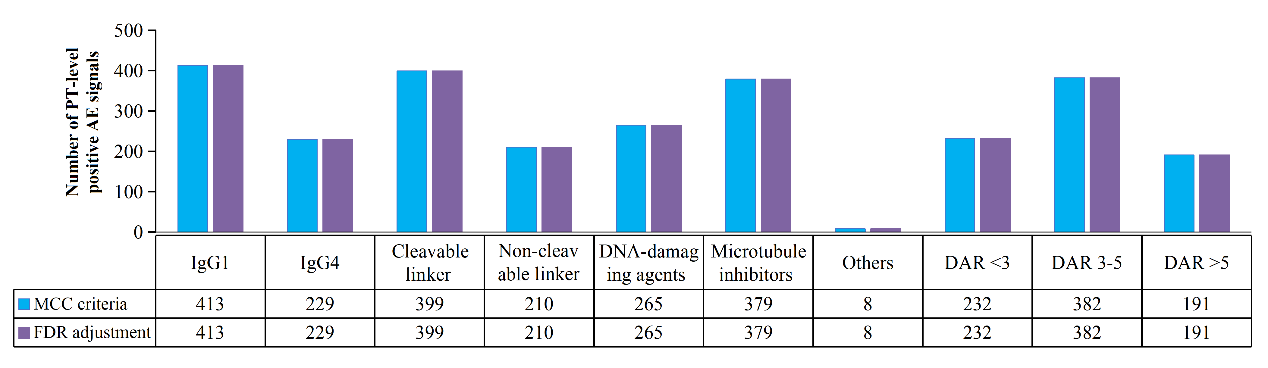


**Supplementary Figure 5.** Upset plot comparing the MCC detection method and the FDR-corrected sensitivity analysis for robust PT-level signals across the four key component types
